# An umbrella review of barriers, facilitators, and interventions for children and youth with disabilities

**DOI:** 10.1093/heapro/daaf237

**Published:** 2026-01-07

**Authors:** Leanne Abungin, Sangeetha Saravanan, Mia Carbone, Kelly Kasper, Jennifer Leo, Carolyn Millar, Kelly P Arbour-Nicitopoulos, Timothy Ross, Amanda Ross-White, Amy Tregubov, Eun-Young Lee

**Affiliations:** School of Kinesiology and Health Studies, Queen’s University, 28 Division St, Kingston, ON, Canada K7L 3N6; School of Kinesiology and Health Studies, Queen’s University, 28 Division St, Kingston, ON, Canada K7L 3N6; Department of Biology, Queen’s University, 116 Barrie St, Kingston, Canada ON K7L 3N6; Program Design & Partnerships, Canadian Tire Jumpstart Charities, 2180 Yonge St, Toronto, ON, Canada M4P 2V8; The Steadward Centre, University of Alberta, Van Vliet Centre, 8831 116 St NW, Edmonton, AB, Canada T6G 1P7; The Steadward Centre, University of Alberta, Van Vliet Centre, 8831 116 St NW, Edmonton, AB, Canada T6G 1P7; Faculty of Kinesiology and Physical Education, University of Toronto, Athletic Centre, 55 Harbord St, Toronto, ON, Canada M5S 2W6; Bloorview Research Institute, Holland Bloorview Kids Rehabilitation Hospital, 150 Kilgour Rd., Toronto, ON, Canada M4G 1R8; Department of Geography and Planning, University of Toronto, 100 George St, Toronto, ON, Canada M5A 2M4; Rehabilitation Sciences Institute, University of Toronto, 500 University Ave., Toronto, ON, Canada M5G 1V7; Bracken Health Sciences Library, Queen’s University, 18 Stuart St, Kingston, ON, Canada K7L 3N6; Canadian Disability Participation Project 2.0 Parent Partner, Canada; School of Kinesiology and Health Studies, Queen’s University, 28 Division St, Kingston, ON, Canada K7L 3N6; Children’s Hospital of Eastern Ontario Research Institute, 401 Smith Rd., Ottawa, ON, Canada K1H 5B2; Department of Sport Industry, Yonsei University, 50 Yonsei-ro, Seoul, 03722, South Korea

**Keywords:** physical activity, inclusion, active play, access, health, wellbeing, ableism, social ecological model

## Abstract

Children and youth with disabilities have fewer opportunities for structured and unstructured active play than their peers without disabilities. This umbrella review explored how active play is defined and perceived among children and youth with disabilities and their adult facilitators, and identified barriers, facilitators, and interventions influencing active play among children and youth with disabilities. A total of 18 review articles were deemed eligible and synthesized guided by the Social Ecological Model (SEM). Among children and youth with disabilities, active play was defined as fun, spontaneous, and intrinsically motivated, with some seeking intense, meaningful experiences. Reviews also reported that children and youth with disabilities value active play as a means to maintain autonomy and connections but also feel excluded from peers. Adult facilitators appear to recognize the importance of active play and adapted activities to meet individual needs. This review has identified barriers and facilitators within the SEM: (i) individual: psychological factors, body function and structure, (ii) interpersonal: decision-making, social support, and socioeconomic status, (iii) organizational: adapted activity demands, program and staff availability, (iv) community: environment characteristics, activities, attitudes, and resources, and (v) public policy: policy gaps. Instructional and behavioral strategies, assistive technologies, and inclusive playground designs are identified as effective play-based interventions for children and youth with disabilities. Findings highlight the need to incorporate the perceptions and experiences of children and youth with disabilities into how active play is conceptualized and operationalized. Reframing active play in research, policy, and practice can promote equity, social inclusion, and health.

Contribution to Health Promotion

- Children and youth with disabilities experience play in diverse, meaningful ways.
- Participation is shaped by multilevel barriers and facilitators: individual, interpersonal, organizational, community, and policy factors.
- Inclusive definitions of play are needed, moving beyond ableist and deficit-oriented perspectives.
- There is a need to incorporate perceptions and experiences of children and youth with disabilities into the conceptualization of play.
- Reframing play in research, policy, and practice for children and youth with disabilities can promote equity, social inclusion, and health.

## Introduction

The United Nations (UN) Convention on the Rights of the Child recognizes that children and youth with disabilities require further attention from researchers, policymakers, and practitioners to fully exercise their right to play (United Nations 2008). Play is an essential component for physical, social, cognitive, and emotional development among all children and youth, regardless of ability. Therefore, it is important to ensure that all children and youth have equal opportunities to engage in play (Nijhof *et al*. 2018, Burriss and Tsao 2002). However, disparities in access to play opportunities are reported for children and youth with disabilities (Ng *et al*. 2023).

Active play refers to a form of play that involves voluntary engagement in activity that is fun and/or rewarding, usually driven by intrinsic motivation, and physical activity of any intensity (Lee *et al*. 2022). Active play generally refers to play activities that involve bodily movement requiring energy expenditure (e.g. running, climbing, jumping from heights, playing tag), distinguishing it from sedentary forms of play (e.g. board games, block play, drawing) that do not involve substantial physical movement. Active play supports physical fitness and gross motor skills, and improved cardiovascular health through sporadic engagement in moderate- to vigorous-intensity physical activity (Brockman *et al*. 2010, Stanley *et al*. 2016, Johnstone *et al*. 2018). It also provides opportunities for children and youth to develop social and cognitive skills including building social relationships, exploration, and problem solving (Burdette and Whitaker 2005, Dennis and Stockall 2015).

Limited, but available data suggest that better understanding of active play among children and youth with disabilities is required (Tremblay *et al*. 2023). Specifically, using a comprehensive evaluation system that assesses physical activity and related indicators for children and youth with disabilities worldwide (Ng *et al*. 2023), the Active Play indicator, evaluated using the benchmark of the percentage of children and youth engaging in active play and unstructured leisure activities for >2 hours/day, was assigned an overall grade of ‘*incomplete*’ due to insufficient data. Among the three countries with available data that participated in the evaluation (Ng *et al.* 2023), the grades were generally low: *D+* for Israel (34%–39% meeting the benchmark), *D* for Finland (27%–33% meeting the benchmark), and *F* for Canada (<20% meeting the benchmark).

Previous literature highlighted that children and youth with disabilities value active play, especially in contexts where they experience success, accomplishment, and meaningful social interaction (Willis *et al*. 2017, Fahy *et al*. 2021, Bhandari 2023). However, a gap persists in the literature regarding how children and youth with disabilities define, perceive, and experience active play, and what barriers and facilitators may impact their meaningful participation. To support active play for children and youth with disabilities, it is essential to first understand how active play is conceptualized, perceived, and carried out within the physical and social contexts that shape its accessibility and inclusion.

The Social Ecological Model (SEM) is a useful framework for understanding the factors influencing an individual’s health (McLeroy *et al*. 1988). The SEM has been previously used to identify factors influencing physical activity participation among children and adults with disabilities (Martin Ginis *et al*. 2016). Identifying evidence on the challenges and opportunities for active play among children and youth with disabilities through the SEM could guide the development of interventions to address barriers at different levels of influence. Building on the previous work (Anaby *et al*. 2013, Askari *et al*. 2015, Martin Ginis *et al*. 2016, Hospodar *et al*. 2023), this review also made use of the International Classification of Functioning, Disability and Health (ICF) model as a guiding framework for thematic synthesis. Therefore, the objectives of this umbrella review were to: (i) explore how active play is defined, perceived, and experienced, (ii) identify barriers and facilitators within the SEM, and (iii) investigate interventions to promote active play among children and youth with disabilities. This review has primarily used person-first language or ‘person with disability’ when discussing play among children and youth with disabilities. Further explanation of this language choice can be found in the Supplementary Text S1.

## Methods

### Approach and eligibility criteria

An umbrella review of existing literature was an appropriate approach to explore this study’s research questions. Umbrella reviews provide an overview of evidence from published systematic reviews and meta-analyses on a broad research topic (Cant *et al*. 2022) (protocol registration: https://doi.org/10.17605/OSF.IO/ZJ6US).

Inclusion criteria included that the reviews must focus on children and youth aged 0–21 years (United Nations n.d.) with physical, mental, developmental, psycho-social disabilities and sensory impairment. Detailed eligibility criteria are available in Supplementary Table S1.

### Database search

A subject librarian (AR-W) at Queen’s University supported the development of this review’s search strategy, which comprised terms associated with children, youth, disability, and active play (see Supplementary Table S2). Briefly, the search strategy combined terms representing a broad range of disability types with terms capturing concepts related to active play (e.g. outdoor active play, leisure activities) and the settings in which it occurs (e.g. playgrounds, parks). This approach ensured the search captured literature on children and youth with diverse disability types across a variety of active play contexts. Searches were conducted in six relevant databases: MEDLINE, Web of Science, EMBASE, CINAHL, and SPORTDiscus in July 2024 with additional hand search conducted in February 2025 to find any review articles that may have been missed in initial database searches.

### Data selection and extraction

Covidence (www.covidence.org) was used to manage references, remove duplicates, and conduct the screening process. Three screeners (LA, MC, SS) independently reviewed titles and abstracts at Level 1 screening and full texts of all review articles at Level 2 screening, with disagreements resolved by consensus or third reviewer (E-YL). Double screening of each article at each level of review ensured accuracy, consistency, and reduced potential bias in the study selection process (inter-rater reliability Cohen's *k* = 0.45 for Level 1 and 0.58 for Level 2 with random agreement rates of 90% at Level 1% and 54% at Level 2). A manual Google Scholar search retrieved two additional review articles (Graham *et al*. 2018, Dabiri Golchin *et al*. 2024). Data extraction of articles was conducted by two reviewers (LA, SS) for study aim, population, type of disability studied, key findings, and acknowledged limitations. A senior researcher (E-YL) reviewed extracted data and resolved discrepancies through discussion.

### Appraisal of the evidence

Two individuals conducted a methodological quality appraisal of both systematic and scoping reviews using the Joanna Briggs Institute (JBI) Critical Appraisal Checklist for Systematic Reviews and Research Syntheses. The JBI checklist consisted of 11 items that assess the methodological quality of the reviews and was conducted independently by pairs or reviewers.

### Data synthesis

Evidence was synthesized thematically in NVivo, a qualitative data analysis software, using the existing analytic frameworks to guide coding and theme development (Braun and Clarke 2012, Martin Ginis *et al*. 2016, Byrne 2022). The first author (LA) conducted inductive open coding to generate descriptive labels, which were organized into broader categories. When applicable, themes were labeled with ICF terminology (Martin Ginis *et al*. 2016). Perceptions captured how children and youth with disabilities and their adult facilitators understood active play, while experiences reflected the actual participation of children and youth with disabilities. During the thematic analysis process, perceptions were coded as descriptions or interpretations of what active play meant, while experiences referred to accounts from children and youth that detailed how they actually engaged in active play across various contexts. Followed by this process, a deductive approach using the SEM framework (McLeroy *et al*. 1988) guided the mapping of themes according to the five SEM levels. Themes were refined and organized through thematic mapping to address the research questions.

## Results

### Study selection

**Figure 1:**
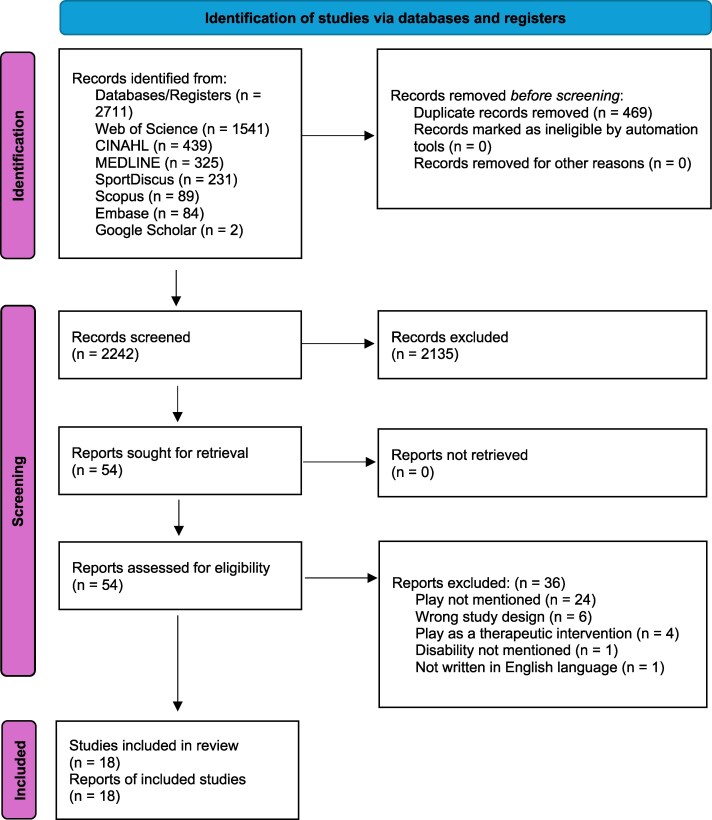
PRISMA flowchart of the study selection process.

Initial database searches identified 2711 articles, including 469 duplicates, resulting in 2242 articles for screening (Supplementary Table S3). The first stage of screening led to the exclusion of 2135 articles based on title and abstract content, leaving 107 articles for full-text review. Of these, 54 full-text articles were assessed in further detail for eligibility, and 16 articles were found to meet inclusion criteria (Fig. 1). One article (Pashmdarfard *et al*. 2021) extended beyond the target age range (0–21 years) but was included due to its relevance to play experiences among children and youth with disabilities. The article did not report the exact mean age; however, most participants ranged between 1.5 and 18 years, with three participants aged 21–24 years. Two review articles (Graham *et al*. 2018, Dabiri Golchin *et al*. 2024) were found through hand searching and added. As a result, a total of 18 review articles were included for review.

### Study characteristics

A total of 18 review studies were included, publication year spanning from 2007 to 2024 (systematic review, n = 10; scoping review, n = 8). The studies encompassed children and youth with disabilities aged 0–24 years with a diverse range of disabilities. Notably, this age range extended slightly beyond the 0–21 years inclusion criteria. None of the studies had reported on mental health disability. Detailed description of each included review is available in Table 1.

**Table 1:** Descriptive summary of the included reviews (*N* = 18).

Author (year)	Search databases	Key concepts	Review type^a^	No. of articles	Type of disability included	Age group	SEM^b^
Anaby *et al*. (2013)	CINAHL, MEDLINE, SSCI, Geobase, EMBASE, and Measures	Terms related to environment, participation “leisure”, “recreation”, “play”), disability, and children	2	31	Cerebral palsy (*n* = 17), physical disabilities (*n* = 6), acquired brain injury (*n* = 3), autism (*n* = 3), down syndrome (*n* = 2)	6–14 years	2, 3, 4, 5
Askari *et al*. (2015)	CINAHL, EMBASE, MEDLINE, and PsychINFO	Terms related to participation (‘leisure’, ‘recreation’, ‘play’), ASD, and children	2	16	ASD, PDD, Asperger syndrome, Kanner syndrome	5–17 years	1, 2, 3, 4, 5
Brown *et al*. (2021)	Medline, PsycINFO, CINAHL, EMBase, ERIC and Scopus	Terms related to playgrounds	2	35	All types of disability included	Not indicated	4, 5
Coussens *et al*. (2022)	MEDLINE and Web of Science	Terms related to participation, parents, children and developmental disability	2	13	ADHD, developmental coordination disorder, ASD	0–6 years	1, 2
Crawford *et al*. (2014)	CINAHL; PubMed; Scopus; PsycINFO; Physical Education Index; ERIC; CBCA Education; and ProQuest Education Journals; and Dissertations and Theses	Terms related to child, disability, play, inclusion, and day care	1	9	Physical disability	2.5–5 years	2, 3
Dabiri Golchin *et al*. (2024)	MEDLINE, CINAHL, Central, ERIC, Scopus, and EMBASE	Terms related to play, children with disabilities, and assistive technology	2	31	All types of disability included	1–20 years	1, 3
Gately *et al*. (2023)	Academic Search Complete/EBSCO, CINAHL/EBSCO, Education Research Complete/EBSCO, ERIC, OTseeker, and PubMed	Terms related to playground, Inclusive, accessible, play, child, and disability	1	9	All types of disability included	3–12 years	1, 4
Graham *et al*. (2018)	CINHAL, PsycINFO, AMED, MEDLINE, EMBASE and ERIC	Terms related to child, youth, physical disability, and play	1	13	Physical disability	0–18 years	1
Hospodar *et al*. (2023)	Medline, CINAHL, PsycNET, and Web of Science	Terms related to toy car and ‘ride on’ car	1	23	All types of disability included	7–21 months	3
Huus *et al*. (2021)	PsycINFO, MEDLINE, CINAHL, PubMed, ERIC and Africa Wide Information	Terms related to children, youth, disability, long-term health condition, and participation	2	17	All types of disability included	0–21 years	1,2,3,4,5
Jung and Sainato (2013)	PsycINFO and ERIC	Terms related to autism, play, play skills, and play intervention	1	26	Autism	0–8 years	1, 3
Kuhaneck *et al*. (2020)	PubMed, PsycINFO, CINAHL, OTDBase, and the Cochrane Database	Terms related to autism and play	1	20	Autism, ASD	3–18 years	3
Laverdure and Beisbier (2021)	MEDLINE, PsycINFO, CINAHL, ERIC, OTseeker, and Cochrane Database	Terms related to children, youth, activities of daily living, and intervention	1	23	All types of disability included	5–21 years	3
Luckett *et al*. (2007)	PsychInfo	Terms related to autism, play, and applied behavior	1	47	Autism	Not indicated	1
Morgenthaler *et al*. (2023)	Academic Search Complete, Avery Index to Architectural Periodicals, CINAHL, MEDLINE, PsycINFO, Scopus, and Web of Science	Terms related to playground, outdoor play, and child	2	51	All types of disability included	0–12 years	1, 4
Pashmdarfard *et al*. (2021)	MEDLINE, CINAHL, PsycINFO, and Embase	Terms related to participation, cerebral palsy, children, active daily living, education, play, leisure, social participation, rest/sleep, work, leisure, occupation, and mobility	1	31	Cerebral palsy	0–24 years	1
Sterman *et al*. (2016)	CINHAL, MEDLINE, Web of Science, ERIC, Scopus, PsycINFO and SocINDEX	Terms related to child, disability, caregiver, play, and outdoors	1	11	Developmental disability	6–12 years	1, 2, 4
Therrien *et al*. (2022)	ProQuest, ERIC, and Child Development and Adolescent Studies	Terms related to playground, disability, autism, and AAC	2	10	Children with AAC needs	0–13 years	1, 2, 4

^a^Type of review: 1 = Systematic review; 2 = Scoping review.

^b^Socioecological Model (SEM) level: 1 = Individual; 2 = Interpersonal; 3 = Organization; 4 = Community; 5 = Public policy.

*n* represents the number of studies in the review that included a specific disability type.

AAC, Augmentative and alternative communication; ADHD, Attention deficit hyperactivity disorder; ASD, Autism spectrum disorder; CBCA Education, Canadian Business & Current Affairs Education; CINAHL, Cumulated Index in Nursing and Allied Health Literature; Embase, Excerpta Medica Database; ERIC, Education Resources Information Center; MEDLINE, Medical Literature Analysis and Retrieval System Online; OTDBase, Occupational Therapy Journal Literature Search Service; OTseeker, Occupational Therapy Systematic Evaluation of Evidence; PDD, Pervasive developmental disorders; SSCI, Social Science Citation Index.

### Quality appraisal of articles

The 10 systematic reviews and eight scoping reviews were assessed using the JBI critical appraisal checklist (Supplementary Table S4a for systematic reviews and Supplementary Table S4b for scoping reviews). All 10 systematic reviews adhered to the JBI checklist, particularly in reporting review objectives, eligibility criteria, and synthesis methods; however, seven reviews did not specify whether data extraction and/or quality appraisal were performed independently by two or more reviewers. Similarly, all scoping adhered to the JBI checklist. All scoping reviews included a clearly defined rationale, detailed search strategy, thorough synthesis of results, and a concise summary of the evidence.

### Definition, perception, and experiences of active play

Among the included 18 review studies, three studies presented a definition of active play (Luckett *et al*. 2007, Graham *et al*. 2018, Morgenthaler *et al*. 2023), seven studies discussed perceptions of active play among children and youth with disabilities and their adult facilitators (Crawford *et al*. 2014, Sterman *et al*. 2016, Graham *et al*. 2018, Brown *et al*. 2021, Coussens *et al*. 2022, Gately *et al*. 2023, Morgenthaler *et al*. 2023). Six studies discussed experiences of active play from children and youth with disabilities (Anaby *et al*. 2013, Askari *et al*. 2015, Graham *et al*. 2018, Huus *et al*. 2021, Therrien *et al*. 2022, Gately *et al*. 2023). Detailed description is also available in Supplementary Table S5.

#### Definition of active play

Three of the included reviews provided similar definitions of active play among children and youth with disabilities, though there was variation in their preference for *intense* play opportunities (Luckett *et al*. 2007, Graham *et al*. 2018, Morgenthaler *et al*. 2023). In general, active play was defined as a positive experience that is fun, self-directed, and intrinsically motivated, with definitions reported either by children and youth with disabilities themselves, adult facilitators, and/or researchers. Specifically, children and youth (0–18 years) with physical disabilities described active play as a positive, fun, and enjoyable experiences (Graham *et al*. 2018). Active play was defined as intrinsically motivated and spontaneous among children with autism, providing them the freedom to engage in an activity without external rewards (Luckett *et al*. 2007). Likewise, children and youth (0–12 years) with disabilities expressed a preference for active play that was fun and self-directed, while also seeking more intense play experiences (Morgenthaler *et al*. 2023). Furthermore, children and youth with disabilities indicated a desire to experience a diversity of intense and novel play opportunities, particularly activities involving high-intensity movements and/or sensory experiences (Morgenthaler *et al*. 2023).

#### Perceptions of play

Three reviews discussed how children and youth with disabilities perceive active play as important for exercising decision-making and fostering social connections (Graham *et al*. 2018, Gately *et al*. 2023, Morgenthaler *et al*. 2023). For example, children and youth with disabilities (0–18 years) often perceived active play as a valuable opportunity to make decisions on their play and to connect with peers. Further, children (0–12 years) with disabilities reported their desire to make their own decisions about what to play, by finding suitable challenges, adapting the environment to their play, and to have uninterrupted moments to unfold their own play (Morgenthaler *et al*. 2023). In two reviews, children and youth (0–18 years) with disabilities valued active play, both when done independently or with others (peers or adults) (Graham *et al*. 2018, Morgenthaler *et al*. 2023). In particular, those with physical disabilities highlighted the importance of playing with peers without disabilities, as they expressed enjoyment in the competitive aspects of active play and valued the opportunity to engage in these experiences alongside others (Graham *et al*. 2018).

In four reviews, adult facilitators discussed the importance of active play in promoting the health and well-being of children and youth with disabilities, and actively considered their unique needs and preferences when facilitating play (Crawford *et al*. 2014, Sterman *et al*. 2016, Brown *et al*. 2021, Coussens *et al*. 2022). For example, caregivers of early years children (0–6 years) with developmental disabilities aimed to encourage a physically active and independent lifestyle, taking into account factors such as the need for additional support in active play activities (Coussens *et al*. 2022). Further, caregivers of children (6–12 years) with developmental disabilities considered the intrinsic factors of their children when determining whether outdoor active play in a particular setting is appropriate (Sterman *et al*. 2016). This consideration was also reflected in findings where caregivers reported that children with disabilities often perceived elevated play structures on playgrounds as more enjoyable than ground-level (Brown *et al*. 2021). Likewise, adults supervising active play in daycare centres reported considering the specific needs and preferences of early years children (2.5–5 years) with physical disabilities when facilitating play activities (Crawford *et al*. 2014).

#### Experiences of play

Four reviews reported challenges facing children and youth with disabilities when playing with peers (Askari *et al*. 2015, Graham *et al*. 2018, Therrien *et al*. 2022, Gately *et al*. 2023). Children and youth (5–17 years) with autism spectrum disorder (ASD) often engaged in solitary play activities due to sensory sensitivities and difficulties with social interaction (Askari *et al*. 2015). Similarly, children (3–12 years) with mobility impairments encountered physical barriers when accessing playgrounds, leading to feelings of separation from peers (Gately *et al*. 2023). Caregivers of children and youth (0–18 years) with physical disabilities reported their child had to seek their permission before engaging in active play, often requiring their approval to participate in play activities (Graham *et al*. 2018). For children (0–13 years) with AAC requirements, communication challenges hindered their active participation in peer-based activities, often resulting in them observing their peers rather than engaging directly (Therrien *et al*. 2022).

Five reviews reported how children and youth with disabilities experienced active play differently from peers without disabilities due to social and environmental factors (Anaby *et al*. 2013, Graham *et al*. 2018, Huus *et al*. 2021, Therrien *et al*. 2022, Gately *et al*. 2023). Although children and youth (0–21 years) with disabilities expressed a desire for spontaneous and self-directed active play, their quality of participation often depended on adult assistance and appropriate adaptations or equipment (Anaby *et al*. 2013, Graham *et al*. 2018, Huus *et al*. 2021). When playing with their peers, children and youth (0–18 years) with physical disabilities often reported that assuming the role of an observer rather than an active participant, as they had to adapt their play to align with their peers without disabilities or seek permission to join active play activities (Graham *et al*. 2018). Likewise, children (0–13 years) with augmentative and alternative communication (AAC) needs reported observing their peers during active play, but not actively participating (Therrien *et al*. 2022).

### Barriers and facilitators of play within the SEM

**Figure 2:**
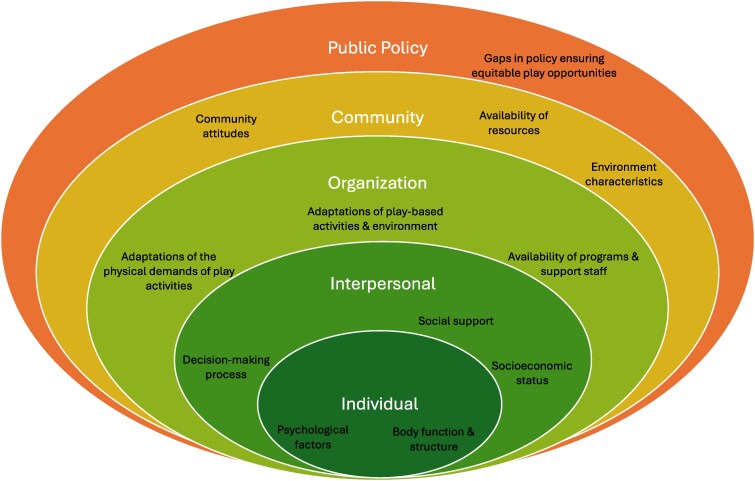
Identified themes of active play among children and youth with disabilities within the social ecological model.

Ten reviews identified facilitators and barriers to play participation, opportunities, and engagement. Facilitators and barriers to play for children and youth with disabilities existed across multiple layers of the SEM framework, including individual, interpersonal, organizational, community, and public policy domains (Fig. 2; Supplementary Table S6). For each layer, facilitators and barriers were organized by themes.

#### Individual factors

At the individual level, active play barriers and facilitators were categorised into the following themes: psychological factors and body function and structure. Seven reviews identified psychological factors, including autonomy, attitudes, beliefs, self-perception, and emotional states as influential to play participation among children and youth with disabilities (Crawford *et al*. 2014, Sterman *et al*. 2016, Graham *et al*. 2018, Brown *et al*. 2021, Huus *et al*. 2021, Therrien *et al*. 2022, Morgenthaler *et al*. 2023).

##### Psychological factors

Three reviews identified that fostering independence and honoring personal preferences supported autonomy in play, which enhanced active play experiences for children and youth with disabilities (Crawford *et al*. 2014, Brown *et al*. 2021, Morgenthaler *et al*. 2023). Autonomy, the ability to make independent decisions, was particularly important for children and youth with disabilities as it supported their agency and participation in play (Crawford *et al*. 2014, Morgenthaler *et al*. 2023). The reviews also highlighted the importance of allowing children to self-select active play activities that aligned with their own interests and preferences, which helps foster greater independence and enjoyment (Brown *et al*. 2021, Morgenthaler *et al*. 2023). Personal preferences for types of play and play partners influenced the quality of play engagement, with children with disabilities (0–12 years) expressing a desire for opportunities to make independent decisions on how they play (Crawford *et al*. 2014, Morgenthaler *et al*. 2023).

Four reviews identified that attitudes, beliefs, self-perception, and emotional states can impact play participation among children and youth with disabilities (Sterman *et al*. 2016, Graham *et al*. 2018, Huus *et al*. 2021, Therrien *et al*. 2022). Children (6–12 years) with disabilities were more likely to participate in play when they perceived it as meaningful and had positive inner-drive (Sterman *et al*. 2016). Conversely, a lack of motivation, negative emotions and behavioral challenges had hindered participation among children (0–13 years) with AAC needs (Therrien *et al*. 2022). Feelings of frustration and exclusion arose when children and youth (0–21 years) with disabilities encountered physical limitations or senses that others did not want to play with them, contributing to reduced engagement in play (Graham *et al*. 2018, Huus *et al*. 2021).

##### Body function and structure

Five reviews identified the body functions and physiology of a child, including the nature of their disability, motor and communication skills, and cognitive function, as factors influencing their participation and engagement in play (Askari *et al*. 2015, Sterman *et al*. 2016, Brown *et al*. 2021, Pashmdarfard *et al*. 2021, Therrien *et al*. 2022). The nature of disability (i.e. severity of disability and clinical symptoms) influenced the ability of children and youth (5–17 years) with disabilities to participate in play and determined the level of support required to facilitate their play (Askari *et al*. 2015, Sterman *et al*. 2016). Motor impairments often hindered engagement and limited opportunities for social interaction during play among children and youth (5–17 years) with ASD (Askari *et al*. 2015). Likewise, children (0–13 years) with AAC needs often faced challenges in interacting with their peers, which lead them to observe play rather than actively participating in it (Therrien *et al*. 2022).

#### Interpersonal factors

Themes identified at the interpersonal level included decision-making process in facilitating active play, social support, and influence of socioeconomic status.

##### Decision-making process in facilitating active play

Two reviews identified adult facilitators as important to enabling and restricting active play opportunities for children and youth with disabilities, with their decision-making process shaped by personal knowledge, preferences, and perceptions of play (Anaby *et al*. 2013, Sterman *et al*. 2016). Prior experiences of adult facilitators and their knowledge of disability-friendly opportunities for outdoor active play influenced children and youth’s decisions regarding play (Sterman *et al*. 2016). Recreation and cultural values and preferences of family members were significantly associated with the intensity in which children and youth with disabilities participate in active play (Anaby *et al*. 2013). Finally, adult facilitators who perceived active play as important and beneficial to child and youth health and well-being were more likely to seek opportunities for them to engage in active play(Sterman *et al*. 2016).

##### Social support

Four reviews found that having social support promoted play participation and enhanced the inclusivity of active play experiences for children and youth with disabilities (Crawford *et al*. 2014, Askari *et al*. 2015, Huus *et al*. 2021, Gately *et al*. 2023). This social support included family, peers, and others (e.g. teachers), all of whom contributed to child and youth active play experiences. Families that provided support for their child’s play and encouraged them to be physically active were found to facilitate play among children and youth (0–21 years) with disabilities (Huus *et al*. 2021). Social support from friends and classmates without disabilities facilitated participation in active play and recreational activities among children and youth (2.5–17 years) with disabilities (Crawford *et al*. 2014, Askari *et al*. 2015). In contrast, a lack of support from peers and teachers was a significant barrier to active play participation (Gately *et al*. 2023).

##### Socioeconomic factors

Four reviews identified socioeconomic factors such as time and financial constraints as significant barriers hindering children and youth with disabilities from participating in active play (Anaby *et al*. 2013, Askari *et al*. 2015, Sterman *et al*. 2016, Pashmdarfard *et al*. 2021). Parents reported being unable to supervise their children (6–12 years) with disabilities or allow them to play outdoors due to limited time (Sterman *et al*. 2016). Time constraints were also mentioned as a barrier among parents and their children and youth (6–14 years) with disabilities as they often had to navigate inaccessible environments where active play took place (Anaby *et al*. 2013). This included challenges such as limited accessible transportation options. Financial considerations influenced decisions on whether to permit their child/youth to participate in outdoor active play opportunities or to transport them to unstructured active play activities (Askari *et al*. 2015, Sterman *et al*. 2016).

#### Organization factors

Three themes identified at the organization level included: adaptation of the physical demands of active play activities, play-based activity and environment adaptations, as well as the availability of programs and support staff to support their active play.

##### Adaptations for the physical demands of active play

Four reviews found that motor assistive devices and AAC devices fostered meaningful active play participation among children and youth with disabilities by adapting the physical demands of activities (Huus *et al*. 2021, Laverdure and Beisbier 2021, Hospodar *et al*. 2023, Dabiri Golchin *et al*. 2024). Motorized ride-on cars (MROC) and power mobility devices increased opportunities for children and youth with disabilities to engage in active play activities with peers (Hospodar *et al*. 2023, Dabiri Golchin *et al*. 2024). Children and youth (0–18 years) with physical disabilities generally viewed their assistive equipment positively, recognizing its role in facilitating active play and that a lack of appropriate adaptations hindered their participation (Graham *et al*. 2018).

##### Adaptations for play-based activities and environments

Four reviews identified the importance of adapting play activities and the play environment, in promoting inclusive play experiences for children and youth with disabilities (Crawford *et al*. 2014, Kuhaneck *et al*. 2020, Brown *et al*. 2021, Hospodar *et al*. 2023). Adapting activities to reduce motor demands and sensory sensitivities enabled greater participation and fostered inclusive active play among children and youth (0–18 years) with disabilities (Crawford *et al*. 2014, Kuhaneck *et al*. 2020, Hospodar *et al*. 2023). Likewise, modifications such as altering where active play occurs, adjusting sensory aspects, or providing visual supports enhanced play performance among children and youth (3–18 years) with autism (Kuhaneck *et al*. 2020). Activities not adapted to take into account an individual’s disability were a barrier to participation among children and youth (0–21 years) with disabilities (Huus *et al*. 2021). Adapting play spaces such as playgrounds also enhanced active play experiences for children and youth (3–18 years) with disabilities (Kuhaneck *et al*. 2020, Brown *et al*. 2021). For example, Brown *et al.* (2021) highlighted the importance of incorporating sensory elements into playgrounds and ensuring their thoughtful distribution throughout, to enhance engagement and prevent overstimulation.

##### Availability of programs and support staff

Three reviews identified the importance of organizational programs and trained support staff in facilitating active play and recreation participation among children and youth with disabilities (Anaby *et al*. 2013, Crawford *et al*. 2014, Askari *et al*. 2015). Schools had promoted active play participation during school hours through adult facilitators (e.g. teachers), and through organized after-school activities (Crawford *et al*. 2014, Askari *et al*. 2015). In a childcare setting, teachers facilitated play among early years children (2.5–5 years) with physical disabilities by tailoring active play activities to the specific needs and/or preferences of a child as well as providing a space for children to engage in peer play (Crawford *et al*. 2014). In contrast, limited participation of children and youth (6–14 years) with disabilities was linked to the lack of personal assistance, specialists, and other supporting staff (Anaby *et al*. 2013).

#### Community factors

At the community level, factors influencing active play were organized into the following themes: environment characteristics, negative community attitudes, and community resource availability.

##### Environmental characteristics

Six reviews identified how physical aspects of communities’ environments influenced the play experiences of children and youth with disabilities (Anaby *et al*. 2013, Askari *et al*. 2015, Sterman *et al*. 2016, Brown *et al*. 2021, Therrien *et al*. 2022, Gately *et al*. 2023). Within community natural environments, favorable outdoor conditions and availability of community parks encouraged children and youth with disabilities to engage in active play (Anaby *et al*. 2013, Askari *et al*. 2015). Playgrounds are reported as crucial built spaces that offered valuable active play opportunities, but they often include barriers to active play for children and youth (5–17 years) with disabilities. Inclusive playgrounds enhanced active play participation among children and youth with disabilities, especially when they were involved in the design process (Brown *et al*. 2021). However, it was also suggested that many children with disabilities still encountered physical barriers to playgrounds labeled as accessible, often requiring adults to provide physical assistance (Sterman *et al*. 2016, Therrien *et al*. 2022, Gately *et al*. 2023). Physical inaccessibility of playgrounds and travel to playgrounds limited the ability of children and youth with disabilities to participate in active play (Sterman *et al*. 2016, Therrien *et al*. 2022).

##### Community attitudes

Four reviews identified negative community attitudes as a barrier to participation for children and youth with disabilities (Anaby *et al*. 2013, Askari *et al*. 2015, Sterman *et al*. 2016, Huus *et al*. 2021). Exposure to stigma and bullying was commonly reported as hindering participation among children and youth with cerebral palsy (6–14 years) and ASD (5–17 years) (Anaby *et al*. 2013, Askari *et al*. 2015). Children and youth (0–21 years) with disabilities were also reported to experience moments during active play when they were not treated as equals by peers without disabilities (Huus *et al*. 2021). For children (6–12 years) with developmental disabilities, prevailing social attitudes toward the abilities of their child affected the decision-making of caregivers on outdoor active play (Sterman *et al*. 2016).

##### Availability of community resources

Four reviews identified similar barriers to active play, including limited access to and knowledge on available community-based programs, and insufficient transportation to play spaces (Anaby *et al*. 2013, Askari *et al*. 2015, Huus *et al*. 2021, Therrien *et al*. 2022). Families of children (0–13 years) with AAC needs often encountered limited access to community-based programs and challenges in transporting their child to active play-related activities (Therrien *et al*. 2022). Similarly, families of children and youth (6–14 years) with cerebral palsy and children and youth (5–17 years) with ASD, experienced barriers to their participation due to limited accessible transportation, community programs, and access to information on available community resources (Anaby *et al*. 2013).

#### Public policy factors

##### Gaps in policy ensuring equitable play opportunities

At the public policy-level, gaps in public policy concerning equitable active play opportunities for children and youth with disabilities were identified as a key theme. While public policies significantly impact accessibility to meaningful active play opportunities for children and youth with disabilities, four reviews identified persistent gaps in policy implementation and the equity they provide (Anaby *et al*. 2013, Askari *et al*. 2015, Huus *et al*. 2021, Therrien *et al*. 2022). Exclusionary elements within policies often restricted active play access by failing to mandate necessary accommodations for children and youth with disabilities. Furthermore, insufficient funding and services for accessible play further hindered participation, as families of children and youth with disabilities frequently faced challenges in accessing programs and transporting their child to and from active play.

### Effectiveness of play-based interventions

Seven reviews examined the effectiveness of play-based interventions for children and youth with disabilities (Supplementary Table S7). The effectiveness of instructional and behavioral strategies, assistive technologies, and inclusive playground designs has been evaluated on how they enhance active play participation.

#### Teaching play skills and behaviors

Two reviews evaluated the effectiveness of instructional and behavioral approaches for teaching play skills to children and youth with disabilities (Luckett *et al*. 2007, Jung and Sainato 2013). These instructional and behavioral approaches directly address individual- and interpersonal-level barriers, including children and youth with disabilities’ challenges in initiating play, social communication, and sustained peer engagement. Their findings suggest that video and live modeling are effective observational learning strategies for children (0–8 years) with autism, as they promoted skill acquisition. Pivotal response training (PRT), an evidence-based intervention grounded in applied behavior analysis to enhance social communication skills among individuals with ASD (Lei and Ventola 2017), enhanced play and social interactions by incorporating child interests into naturalistic instructional settings (Jung and Sainato 2013). Behavioral interventions that rely on the motivating nature of play activities were found to be highly effective but also indicated that the processes underlying generalization, how children apply learned skills in new situations, were not well understood (Luckett *et al*. 2007).

#### Assistive technologies

Two reviews included studies that examined how assistive technologies targeting improved mobility and communication can increase active play participation for children and youth with disabilities (Hospodar *et al*. 2023, Dabiri Golchin *et al*. 2024). Assistive technologies can mitigate barriers at the interpersonal- and organizational-level by addressing mobility- and communication-related challenges, including limited physical access to play spaces and difficulty participating in or communicating during peer play. MROCs, as a means to support active mobility and participation, were recently shown to improve play participation among children with physical disabilities (Hospodar *et al*. 2023, Dabiri Golchin *et al*. 2024). Children and youth (1–20 years) with disabilities viewed the use of mobility devices, such as MROCs, in their active play as enjoyable (Dabiri Golchin *et al*. 2024). However, the perceived barriers related to outdoor active play environments were reported to inhibit the use of MROCs and other assistive technologies. The use of communication devices (e.g. AAC) enhanced active play participation, collaboration, and communication among children and youth with AAC needs, enabling them to engage more effectively in play with peers (Dabiri Golchin *et al*. 2024). Overall, these findings support the use of assistive technologies such as MROCs and AACs to increase active play participation among children with physical disabilities.

#### Inclusive playground design

One review discussed evidence-informed recommendations on inclusive playground design, highlighting how future playgrounds can be designed to enhance usability and inclusiveness for all children (Brown *et al*. 2021). The study outlined 13 evidence-informed recommendations and one promising practice that consider playground entry, play components to promote inclusive active play, the role of trained staff on playgrounds, and the involvement of families of children with disabilities in the playground design process. These design recommendations directly respond to organizational- and community-level barriers such as inaccessible built environments, limited trained support staff, and limited engagement with users (i.e. children and youth with disabilities) in design processes. Practitioners are encouraged to actively engage children and youth with disabilities, their families, and disability advocates in planning and design processes to help ensure the play environments account for their lived experiences, accessibility requirements, and play preferences.

## Discussion

Findings from this umbrella review suggest that a critical reexamination of traditional conceptualizations of active play is required to ensure these conceptualizations reflect the diverse abilities, perceptions, and experiences, of children and youth with disabilities. This review’s findings suggest that meaningful active play experiences for children and youth with disabilities are influences by interacting influences across multiple SEM levels, with individual, interpersonal, organization, community, and public policy factors collectively shaping perceptions and experiences of play for this population group. This review also highlights how the language used to describe disability influences how active play is conceptualized and understood for children and youth with disabilities.

Our findings align with literature that finds children and youth with disabilities experience active play in distinct and meaningful ways, prompting a reconsideration of what constitutes meaningful participation in active play. Adaptations through technology, the play activity itself, or modified environments were identified as important for enabling children and youth with disabilities to participate meaningfully in active play (Crawford *et al*. 2014, Kuhaneck *et al*. 2020, Brown *et al*. 2021, Hospodar *et al*. 2023, Dabiri Golchin *et al*. 2024). This is likely because such adaptations help remove physical and sensory barriers, often being perceived as an integral part of how children and youth with disabilities participate and interact in play environments. Also, recent studies beyond this review noted that children and youth with disabilities participate in active play meaningfully both as an active participant and observer (Kuhaneck *et al*. 2020, Fahy *et al*. 2021). Although being an observer is a passive form of play, recent literature, along with our findings, challenge this assumption for children and youth with disabilities (Graham *et al*. 2018, Kuhaneck *et al*. 2020). Health benefits are likely greater when children and youth with disabilities engage in active play directly, given their reported positive feelings when peers invite and include them in play activities (Graham *et al*. 2018). However, play through observation can be beneficial, as it is typically self-directed with autonomy; therefore, can be acknowledged as a valid and meaningful form of active participation (Kuhaneck *et al*. 2020). This perspective invites us to reconsider what meaningful participation in active play entails for children and youth with disabilities.

Conventional definitions of active play often overlook the varied ways children and youth with disabilities participate. Both self-directed and structured forms of active play are valued for supporting participation of children and youth with disabilities, with structured and guided active play recognized as particularly beneficial for those requiring additional support (Crawford *et al*. 2014, Sterman *et al*. 2016, Brown *et al*. 2021, Coussens *et al*. 2022). For example, children and youth with autism reported facing challenges with socializing with their peers during unstructured active play in playground settings (Akers *et al*. 2018, Khatab *et al*. 2024). Adults facilitating guided active play could be suitable in this setting as they can provide physical assistance and support with selecting appropriate activities, and help ensure children’s safety during active play (Toub *et al*. 2016). These findings challenge conventional definitions of active play that emphasize spontaneity and independence with minimal adult supervision, which calls for more inclusive and operational definitions that reflect the diverse ways children and youth with disabilities experience and perceive active play.

Lack of conceptualization and operationalization of active play was noted as a gap in previous studies outside this review, which led to obtaining international consensus on its ontology, taxonomy, and terminology (Lee *et al*. 2022, Lee *et al*. 2024). Play is universally defined as a voluntary engagement in an activity that is fun and/or rewarding and usually driven by intrinsic motivation but also noted that not all active play is necessarily self-directed or intrinsically motivated and nuanced interpretation is needed to consider active play across different contexts. Such definition of active play aligns with how active play was defined across the included studies (Luckett *et al*. 2007, Graham *et al*. 2018, Morgenthaler *et al*. 2023), where intrinsic motivation, enjoyment, and autonomy were emphasized. However, the included studies often expanded on this conceptualization by contextualizing how active play is experienced among children and youth with disabilities. This suggests that the universally accepted definition (Lee *et al*. 2022) may not fully capture the meaning of active play and how active play is experienced among this group, and may require refinement if we are to advance play inclusion for children and youth with disabilities. Specifically, conceptualizing active play primarily around voluntary engagement and intrinsic motivation may inadvertently exclude forms of active play that are facilitated, adapted, or structured, which are common realities for children and youth with disabilities.

Our findings highlight the multiple levels of influence within the SEM that shape active play perception and experiences among children and youth with disabilities. The factors influencing play ranged from individual preferences and functioning to the role of interpersonal relationships, adaptive programs and technologies, inclusive play environments, and design policies. This complex systems perspective highlights that a single SEM level of influence is not sufficient on its own. For example, interventions at the individual level may not effectively enhance meaningful participation in active play without supportive social systems, accessible and inclusive play programs and environments. Our review also found a notable lack of information at the public policy level regarding what influences active play experiences for children and youth with disabilities. This is a concern because policy has been identified as an important factor in shaping the built and social environments conducive to active play for children and youth with disabilities (Shikako-Thomas and Law 2015, Martin Ginis *et al*. 2016). Policy-driven play spaces and community play programs reflect and perpetuate societal values about who does and does not belong in a particular place (Brown *et al*. 2021, Bell 2025). Without effective policies and sufficient resources, efforts to promote inclusive active play remains insufficient, leaving children and youth with disabilities underserved.

The disability language used across the reviews reveals how scholars are conceptualizing disability. While some reviews used person-first language (i.e. stating ‘children and youth with disabilities’), others adopted identity-first language (i.e. stating ‘disabled children and youth’), and they often did so inconsistently. A few reviews, particularly those rooted in a medical perspective, framed disability to emphasize deficits and functional limitations (Askari *et al*. 2015, Brown *et al*. 2021, Pashmdarfard *et al*. 2021). Although medical interventions are important in improving the quality of life for individuals with disabilities, they simultaneously uphold normative assumptions of how bodies are expected to function (Bell 2025). Furthermore, this medicalized framing, including the use of person-first language in some studies, may inadvertently pathologize disability and undermine efforts to address and prevent social and environmental barriers to active play participation (Ross 2013). The implications of such language choices are important as they shape how disability is understood in research and influence whether interventions focus on changing the child or the context in which they play.

This review explored how active play is defined, perceived, and experienced among children and youth with disabilities—a perspective that remains underexplored despite its importance for advancing inclusive active play opportunities and informing policy development. This review followed a robust and transparent methodological approach consistent with umbrella review guidelines. A protocol for this review was pre-registered to enhance transparency and methodological consistency. The use of a comprehensive, librarian-developed search strategy across six databases, supplemented by hand searching, ensured broad coverage of the literature on active play and disability. The involvement of independent reviewers at each stage of screening, data extraction, and quality appraisal helped to strengthen reliability and minimize bias.

Despite its strengths, this review has limitations. First, the inherently broad scope of an umbrella review may limit specificity, which may overlook relevant details presented in individual studies included in each review we considered. Secondly, while the review aimed to centre the perspectives of children and youth with disabilities who directly encounter barriers and facilitators to active play, many of the included reviews reflected perspectives of their adult facilitators, which may have limited the authenticity of how children and youth with disabilities themselves define, perceive, and experience active play. Thirdly, it was difficult to distinguish active play among early years children with disabilities, as there is little known about how active play should be conceptualized and experienced for this age group. Finally, while public policy-level insights emerged, they were underrepresented and often lacked specificity, highlighting a critical gap in the literature regarding active play for children and youth with disabilities.

## Conclusion

This review highlights that children and youth with disabilities perceive and experience active play in diverse and meaningful ways that prompt a reconsideration of how active play is conceptualized. Furthermore, active play for children and youth with disabilities is shaped by different layers of factors within the SEM, each of which can support and/or hinder participation. The reliance on adult facilitation and adaptation support for meaningful participation in active play emphasizes the need to reimagine play beyond historical, non-disabled paradigms and understanding of what constitutes as active play. This reimaging of active play goes beyond addressing physical and social barriers to valuing the presence and diverse perceptions and experiences of children and youth with disabilities. Conceptualizing active play sole in terms of voluntary engagement and intrinsic motivation risks excluding forms of play that are facilitated, adapted, or structured, common realities for children and youth with disabilities. Future research should examine how inclusive definitions of active play can be operationalized within research, policy, and practice to better support participation among children and youth with disabilities. A collective commitment across research, policy, and practice is needed to affirm disability as an integral aspect of an individual, rather than a deficit to be fixed.

## Supplementary Material

daaf237_Supplementary_Data

## Data Availability

The data extracted and analyzed for this review are available in the online Supplementary File. All original data sources are publicly available in the published literature cited in this article.

## References

[daaf237-B1] Akers JS, Higbee TS, Gerencser KR et al An evaluation of group activity schedules to promote social play in children with autism. J Appl Behav Anal 2018;51:553–70. 10.1002/jaba.474 29761491

[daaf237-B2] Anaby D, Hand C, Bradley L et al The effect of the environment on participation of children and youth with disabilities: a scoping review. Disabil Rehabil 2013;35:1589–98. 10.3109/09638288.2012.748840 23350759

[daaf237-B3] Askari S, Anaby D, Bergthorson M et al Participation of children and youth with autism spectrum disorder: a coping review. Rev J Autism Dev Disord 2015;2:103–14. 10.1007/s40489-014-0040-7

[daaf237-B4] Bell SA . A sociological examination of playground marginalization and the importance of inclusive spaces. Footnotes 2025;18. https://journal.lib.uoguelph.ca/index.php/footnotes/article/view/8344

[daaf237-B5] Bhandari R . Creating effective, empowered, and inclusive play in children with disabilities. SHEJ: Disability Rehabilitation Research 2023;1:3–12. https://oro.open.ac.uk/87452/

[daaf237-B6] Braun V, Clarke V. Thematic analysis. In: Cooper H, Camic PM, Long DL (eds.) APA Handbook of Research Methods in Psychology, Vol. 2. Research Designs: Quantitative, Qualitative, Neuropsychological, and Biological. American Psychological Association, 2012, 57–71.

[daaf237-B7] Brockman R, Jago R, Fox KR. The contribution of active play to the physical activity of primary school children. Prev Med 2010;51:144–7. 10.1016/j.ypmed.2010.05.012 20561971 PMC2917007

[daaf237-B8] Brown DMY, Ross T, Leo J et al A scoping review of evidence-informed recommendations for designing inclusive playgrounds. Front Rehabil Sci 2021;2:664595. 10.3389/fresc.2021.664595 36188796 PMC9397725

[daaf237-B9] Burdette HL, Whitaker RC. Resurrecting free play in young children: looking beyond fitness and fatness to attention, affiliation, and affect. Arch Pediatr Adolesc Med 2005;159:46–50. 10.1001/archpedi.159.1.46 15630057

[daaf237-B10] Burriss KG, Tsao L-L. Review of research: how much do we know about the importance of play in child development? Child Educ 2002;78:230–3. 10.1080/00094056.2002.10522188

[daaf237-B11] Byrne D . A worked example of Braun and Clarke’s approach to reflexive thematic analysis. Qual Quant 2022;56:1391–412. 10.1007/s11135-021-01182-y

[daaf237-B12] Cant R, Ryan C, Kelly MA. A nine-step pathway to conduct an umbrella review of literature. Nurse Author Ed 2022;32:31–4. 10.1111/nae2.12039

[daaf237-B13] Coussens M, Van Waelvelde H, Desoete A et al Parent's perspectives on participation of young children with attention deficit hyperactive disorder, developmental coordination disorder, and/or autism spectrum disorder: a systematic review. Dev Med Child Neurol 2022;64:69–70. 10.1111/cch.12735

[daaf237-B14] Crawford SK, Stafford KN, Phillips SM et al Strategies for inclusion in play among children with physical disabilities in childcare centers: an integrative review. Phys Occup Ther Pediatr 2014;34:404–23. 10.3109/01942638.2014.904470 24712842

[daaf237-B15] Dabiri Golchin M, Ripat J, Verdonck M. Assistive technology to facilitate children's play: a scoping review. Disabil Rehabil Assist Technol 2024;19:2419–29. 10.1080/17483107.2023.2298825 38166593

[daaf237-B16] Dennis LR, Stockall N. Using play to build the social competence of young children with language delays: practical guidelines for teachers. Early Child Educ J 2015;43:1–7. 10.1007/s10643-014-0638-5

[daaf237-B17] Fahy S, Delicâte N, Lynch H. Now, being, occupational: outdoor play and children with autism. J Occup Sci 2021;28:114–32. 10.1080/14427591.2020.1816207

[daaf237-B18] Gately KA, Zawadzki AH, Mosley AM et al Occupational injustice and the right to play: a systematic review of accessible playgrounds for children with disabilities. Am J Occup Ther 2023;77:7702205040. 10.5014/ajot.2023.050035 37040103

[daaf237-B19] Graham N, Nye C, Mandy A et al The meaning of play for children and young people with physical disabilities: a systematic thematic synthesis. Child Care Health Dev 2018;44:173–82. 10.1111/cch.12509 28905445

[daaf237-B20] Hospodar CM, Feldner HA, Logan SW. Active mobility, active participation: a systematic review of modified ride-on car use by children with disabilities. Disabil Rehabil Assist Technol 2023;18:974–88. 10.1080/17483107.2021.1963330 34435924 PMC9328769

[daaf237-B21] Huus K, Schlebusch L, Ramaahlo M et al Barriers and facilitators to participation for children and adolescents with disabilities in low- and middle-income countries—a scoping review. Afr J Disabil 2021;10:1–10. 10.4102/ajod.v10i0.771 PMC8008013 33824860

[daaf237-B22] Johnstone A, Hughes AR, Martin A et al Utilising active play interventions to promote physical activity and improve fundamental movement skills in children: a systematic review and meta-analysis. BMC Public Health 2018;18:789. 10.1186/s12889-018-5687-z 29940923 PMC6019649

[daaf237-B23] Jung S, Sainato DM. Teaching play skills to young children with autism. J Intellect Dev Disabil 2013;38:74–90. 10.3109/13668250.2012.732220 23157647

[daaf237-B24] Khatab S, Hijab MHF, Othman A et al Collaborative play for autistic children: a systematic literature review. Entertain Comput 2024;50:100653. 10.1016/j.entcom.2024.100653

[daaf237-B25] Kuhaneck H, Spitzer SL, Bodison SC. A systematic review of interventions to improve the occupation of play in children with autism. OTJR (Thorofare N J) 2020;40:83–98. 10.1177/1539449219880531 31642399

[daaf237-B26] Laverdure P, Beisbier S. Occupation- and activity-based interventions to improve performance of activities of daily living, play, and leisure for cildren and youth ages 5 to 21: a systematic review. Am J Occup Ther 2021;75:7501205050p1–7501205050p24. 10.5014/ajot.2021.039560 33399053

[daaf237-B27] Lee E-Y, de Lannoy L, Li L et al Play, Learn, and Teach Outdoors-Network (PLaTO-Net): terminology, taxonomy, and ontology. Int J Behav Nutr Phys Act 2022;19:66. 10.1186/s12966-022-01294-0 35701784 PMC9199154

[daaf237-B28] Lee E-Y, Shih A-C, Tremblay M. Exploring the world of active play: a comprehensive review of global surveillance and monitoring of active play based on the global matrix data. J Exerc Sci Fit 2024;22:254–65. 10.1016/j.jesf.2024.03.008 38577389 PMC10990752

[daaf237-B29] Lei J, Ventola P. Pivotal response treatment for autism spectrum disorder: current perspectives. Neuropsychiatr Dis Treat 2017:1613–26. 10.2147/NDT.S120710 28790824 PMC5488784

[daaf237-B30] Luckett T, Bundy A, Roberts J. Do behavioural approaches teach children with autism to play or are they pretending? Autism 2007;11:365–88. 10.1177/1362361307078135 17656400

[daaf237-B31] Martin Ginis KA, Ma JK, Latimer-Cheung AE et al A systematic review of review articles addressing factors related to physical activity participation among children and adults with physical disabilities. Health Psychol Rev 2016;10:478–94. 10.1080/17437199.2016.1198240 27265062

[daaf237-B32] McLeroy KR, Bibeau D, Steckler A et al An ecological perspective on health promotion programs. Health Educ Q 1988;15:351–77. 10.1177/109019818801500401 3068205

[daaf237-B33] Morgenthaler T, Schulze C, Pentland D et al Environmental qualities that enhance outdoor play in community playgrounds from the perspective of children with and without disabilities: a scoping review. Int J Environ Res Public Health 2023;20:1763. 10.3390/ijerph20031763 36767130 PMC9913926

[daaf237-B34] Ng K, Sit C, Arbour-Nicitopoulos K et al Global matrix of para report cards on physical activity of children and adolescents with disabilities. Adapt Phys Activ Q 2023;40:409–30. 10.1123/apaq.2022-0111 36963407

[daaf237-B35] Nijhof SL, Vinkers CH, van Geelen SM et al Healthy play, better coping: the importance of play for the development of children in health and disease. Neurosci Biobehav Rev 2018;95:421–9. 10.1016/j.neubiorev.2018.09.024 30273634

[daaf237-B36] Pashmdarfard M, Richards LG, Amini M. Factors affecting participation of children with cerebral palsy in meaningful activities: systematic review. Occup Ther Health Care 2021;35:442–79. 10.1080/07380577.2021.1938339 34191669

[daaf237-B37] Ross T . Advancing Ontario's accessibility: a study of linguistic, discursive, and conceptual barriers. Can J Urban Res 2013;22:126–44. https://www.jstor.org/stable/26193929

[daaf237-B38] Shikako-Thomas K, Law M. Policies supporting participation in leisure activities for children and youth with disabilities in Canada: from policy to play. Disabil Soc 2015;30:381–400. 10.1080/09687599.2015.1009001

[daaf237-B39] Stanley RM, Jones RA, Cliff DP et al Increasing physical activity among young children from disadvantaged communities: study protocol of a group randomised controlled effectiveness trial. BMC Public Health 2016;16:1095. 10.1186/s12889-016-3743-0 27756277 PMC5069890

[daaf237-B40] Sterman J, Naughton G, Froude E et al Outdoor play decisions by caregivers of children with disabilities: a systematic review of qualitative studies. J Dev Phys Disabil 2016;28:931–57. 10.1007/s10882-016-9517-x

[daaf237-B41] Therrien MCS, Barton-Hulsey A, Wong S. A scoping review of the playground experiences of children with AAC needs. Augmentative and Alternative Communication 2022;38:245–55. 10.1080/07434618.2022.2155874 36562096

[daaf237-B42] Toub TS, Rajan V, Golinkoff RM et al Guided play: a solution to the play versus learning dichotomy. In: Geary DC, Berch DB (eds.), Evolutionary Perspectives on Child Development and Education Evolutionary Psychology. Champaign: Springer International Publising, 2016, 117–41.

[daaf237-B43] Tremblay MS, Demchenko I, Reilly JJ et al The future of para report cards on physical activity of children and adolescents with disabilities—a global call for engagement, data, and advocacy. Adapt Phys Activ Q 2023;41:1–8. 10.1123/apaq.2023-0131 37944506

[daaf237-B44] United Nations . Who Are the Youth? n.d. https://www.un.org/en/global-issues/youth#:∼:text=For statistical purposes%2C%20however%2C the,of 15 and 24 years.year (16 September 2025, date last accessed).

[daaf237-B45] United Nations . Convention on the Rights of Persons with Disabilities. 2006. https://www.ohchr.org/en/instruments-mechanisms/instruments/convention-rights-persons-disabilities (16 September 2025, date last accessed).

[daaf237-B46] Willis C, Girdler S, Thompson M et al Elements contributing to meaningful participation for children and youth with disabilities: a scoping review. Disabil Rehabil 2017;39:1771–84. 10.1080/09638288.2016.1207716 27442686

