# Supplementary material for: An umbrella review of barriers, facilitators, and interventions for children and youth with disabilities
Source: Health Promot Int. 2026 Jan 7;41(1):daaf237. doi: 10.1093/heapro/daaf237 (PMC12778329; doi:10.1093/heapro/daaf237)
Supplement: daaf237_Supplementary_Data [file daaf237_supplementary_data.docx]

**An Umbrella Review of Barriers, Facilitators, and Interventions for Children and Youth with Disabilities**

**Supplementary File**: This document contains the supplementary material for the above paper.

Table of Contents

[Supplementary Text 1. Disability language. 2](#_Toc216979709)

[Supplementary Table 1. Review eligibility criteria. 3](#_Toc216979710)

[Supplementary Table 2. Complete search strategy. 5](#_Toc216979711)

[Supplementary Table 3. Database search records. 21](#_Toc216979712)

[Supplementary Table 4a. Critical appraisal for systematic reviews (n = 10). 22](#_Toc216979713)

[Supplementary Table 4b. Critical appraisal for scoping reviews (n = 8). 23](#_Toc216979714)

[Supplementary Table 5. Definition, perception, and/or experiences of active play among children and youth with disabilities and their adult facilitators (n = 10). 24](#_Toc216979715)

[Supplementary Table 6. Facilitators and barriers of active play among children and youth with disabilities (n = 10). 28](#_Toc216979716)

[Supplementary Table 7. Effectiveness of interventions targeting active play among children and youth with disabilities (n = 7). 38](#_Toc216979717)

# **Supplementary Text 1. Disability language.**

This review has primarily used person-first language or “person with disability” when discussing play among children and youth with disabilities. The use of person-first language was informed by its widespread acceptance and predominant use across organizations, legislative frameworks, and academic literature advocating for people with disabilities (Best et al., 2022; Grech et al., 2024). Person-first language was adopted by the UN in the 2006 *Convention on the Rights of Persons with Disabilities* and by national legislations such as the 2019 *Accessible Canada Act* (Accessible Canada Act, S.C. 2019, c. 10., 2019; United Nations, 2006). In addition, scholarly writing style guides such as the *American Psychological Association* recommended writers to use person-first language (Grech et al., 2024).

Person-first language emerged from the disability activism movement, which aimed to empower individuals with disabilities by placing emphasis on their humanity rather than their disability (Best et al., 2022; Crocker & Smith, 2019; Ferrigon, 2019). The language seeks to promote respect for the individual by shifting focus away from their disability. In contrast, identity-first language or “disabled person” acknowledges disability as an integral aspect of the individual’s identity (Best et al., 2022). Rooted in the social model of disability, identity-first language recognizes that disability is a consequence of societal attitudes and environmental barriers that *disable* people (Brinkman et al., 2023). As a result, there is a growing preference for the use of identity-first language among advocates for the disability community as well as those with lived experience. While the authors of this review acknowledge the importance of identity- first language in affirming disability as an important part of an individual’s identity, this review adopts person-first language to maintain clarity and consistency, particularly given the inclusion of review studies encompassing a wide range of disabilities. We recognize the importance of using the preferred language of those with lived experience of disability; however, this can be challenging to determine in a review of secondary sources, where the language used by authors may not reflect the preferences of their participants. Therefore, the term “children and youth with disabilities” was used to reduce potential confusion and ensure consistency throughout the review.

# **Supplementary Table 1. Review eligibility criteria**.

| **Include** | **Exclude** |
| --- | --- |
| **Population** | |
| Age group: 0-21 years  Children and youth with physical disabilities (e.g., Amputation of limb, Spinal Cord Injury), developmental and neurodevelopmental disabilities (e.g., Autism Spectrum Disorders, Cerebral Palsy, Down Syndrome, Muscular Dystrophy), psycho-social disabilities and sensory impairment. Detailed search terms are copied below.   - Disability status may not have been diagnosed in younger age groups; therefore, studies consisting of children and youth with undiagnosed disability status will also be considered. - Co-morbidity with other disabilities that meet the inclusion criteria will be considered as a secondary intervention/exposure of interest. | Children with comorbidities, acute/chronic illness and/or conditions (e.g., cancer, asthma) |
| **Intervention/Exposure** | |
| - Review papers providing information about the definition and/or perception of play among children and youth with disabilities or their parents/guardians/decision makers - Review papers that report on the opportunities for and/or engagement in play among children and youth with disabilities. Detailed search terms are copied below. - Play, active play, and active outdoor play activities that are self-directed and spontaneous | - Play activities that are implemented as a therapeutic intervention - Structured or organized play/outdoor play programs - Resistance training or structured workout |
| - **Comparator/Context** | |
| - Any individual-, interpersonal-, organization-, community-, and public policy-level factors in relation to places and spaces that influence the accessibility, opportunities, or experiences of play for children and youth with disabilities - Any barriers to or facilitators of play in relation to places and spaces among children and youth with disabilities - Any existing interventions aimed at promoting play among children and youth with disabilities by improving places and spaces |  |
| - Any place or space where active play may occur for children and youth with disabilities including, but not limited to, outdoor play, indoor play, play in early learning centers - Any built or natural environment characteristics that could influence active play among children and youth with disabilities | - Studies which use play-based protocols |
| **Review Design** | |
| Systematic review, scoping review, meta-synthesis, realist review, narrative review, critical review, rapid review, integrative review, and other reviews not mentioned. | Grey literature |
| **Publication Type** |  |
| Peer reviewed, refereed review papers | Primary sources, book chapters, commentaries, dissertations, conference proceedings, dissertations, non-peer reviewed sources, opinion letters including protocols. |
| Language: English |  |
| Publication year: No limit |  |
| Context: In-person contexts | Virtual contexts |

# **Supplementary Table 2. Complete search strategy.**

| <https://proxy.queensu.ca/login?url=http://ovidsp.ovid.com?T=JS&NEWS=N&PAGE=main&SHAREDSEARCHID=7Onl6J9X5G0CL7jqssWULGAyXMIQ7BhM511B68Bkl5BqtT299q142QCCEXQnzdB1j>  **Ovid MEDLINE(R) ALL <1946 to August 08, 2024>**  1 ((disabled adj1 pe$) or disab$).mp. or Disabled Persons/ 429447  2 Amputee$.mp. or AMPUTEES/ 8390  3 (hearing adj1 impaired pe$).mp. or Persons with Hearing Impairments/ 3536  4 (developmental adj1 disabilit$).mp. or Developmental Disabilities/ 27687  5 (dependent adj1 ambulation).mp. or Dependent Ambulation/ 232  6 PARAPLEGIA/ or paraplegia.mp. 22964  7 quadriplegia.mp. or QUADRIPLEGIA/ 10398  8 hearing loss.mp. or Hearing Loss/ 83642  9 blindness.mp. or BLINDNESS/ 50928  10 (vision adj1 disorder$).mp. or Vision Disorders/ 31847  11 Mental retardation/ or (((mental adj1 retard) or mentally) adj1 retarded).mp. 63567  12 (mobility adj1 limitation$).mp. or Mobility limitation/ 6460  13 (((self-help adj1 devices) or assistive) adj1 technology).mp. or Self-Help Devices/ 7766  14 (functional adj1 limitation$).mp. 9237  15 (activity adj1 limitation$).mp. 4178  16 (mobility adj1 impairment).mp. 811  17 (vision adj1 impairment$).mp. 3378  18 (hearing adj1 impairment$).mp. 16968  19 (cognitive adj1 impairment$).mp. 102160  20 (intellectual adj1 disabilit$).mp. 76051  21 (participation adj1 limitation$).mp. 189  22 Spinal Cord Injuries/ or SCI.mp. 72284  23 amputation.mp. or AMPUTATION/ 56800  24 cerebral palsy.mp. or Cerebral Palsy/ 33632  25 spina bifida.mp. or Spinal Dysraphism/ 12761  26 multiple sclerosis.mp. or Multiple Sclerosis/ 104892  27 Amyotrophic Lateral Sclerosis/ or ALS.mp. 83036  28 Traumatic brain injury.mp. or Brain Injuries, Traumatic/ 52508  29 BRAIN CONCUSSION/ or concussion.mp. 17746  30 muscular dystrophy.mp. or Muscular Dystrophies/ 34338  31 stroke.mp. or STROKE/ 398742  32 spinal muscular atrophy.mp. or Muscular Atrophy, Spinal/ 8694  33^[[1]](#footnote-1)^ myopathy.mp. or Muscular Diseases/ 45690  34^[[2]](#footnote-2)^ ((sensory adj1 (impairment or limitation)) or "psycho-social disabilit*" or "psychosocial disabilit*").mp. 2622  35^[[3]](#footnote-3)^ exp Child/ or exp infant/ or adolescent/ or exp pediatrics/ or child, abandoned/ or exp child, exceptional/ or child, orphaned/ or child, unwanted/ or minors/ or Young Adult/ or (pediatric* or paediatric* or (child* not childbearing) or newborn* or congenital* or infan* or baby or babies or neonat* or pre-term or preterm* or "premature birth*" or NICU or preschool* or pre-school* or kindergarten* or kindergarden* or "elementary school*" or "nursery school*" or ("day care*" not adult*) or schoolchild* or toddler* or boy or boys or girl* or "middle school*" or pubescen* or juvenile* or teen* or youth* or "high school*" or adolesc* or pre-pubesc* or prepubesc*).mp. or (college or universit* or post-secondary).ab,ti. or (child* or adolesc* or pediat* or paediat*).jn. 6188257  36 Parks, Recreational/ or (outside or outdoor* or "play ground*" or playground* or yard* or (school adj ground*) or "green space*" or greenspace* or "blue space*" or bluespace* or wilderness or natur* or forest* or park or parks or "back yard*" or backyard* or environment* or land-based or water-based or beach or bush or gym or gyms).ti,ab. 3004606  37 exp Exercise/ or exp "Play and Playthings"/ or ((physical adj activity) or active or exercis* or sport* or leisure or recreation* or adventur* or movement or play* or game or games or gym or gyms).ti,ab. 3569181  38 or/1-34 1535996  39 35 and 36 and 37 and 38 5614  40 limit 39 to english language 5341  41 (systematic review or meta-analysis).pt. 357325  42 meta-analysis/ or systematic review/ or systematic reviews as topic/ or meta-analysis as topic/ or "meta analysis (topic)"/ or "systematic review (topic)"/ or exp technology assessment, biomedical/ or network meta-analysis/ 399571  43 (((systematic* or scoping) adj3 (review* or overview*)) or (methodologic* adj3 (review* or overview*))).ti,ab,kf. 397496  44 ((quantitative adj3 (review* or overview* or synthes*)) or (research adj3 (integrati* or overview*))).ti,ab,kf. 17882  45 ((integrative adj3 (review* or overview*)) or (collaborative adj3 (review* or overview*)) or (pool* adj3 analy*)).ti,ab,kf. 43450  46 (data synthes* or data extraction* or data abstraction*).ti,ab,kf. 46466  47 (handsearch* or hand search*).ti,ab,kf. 11698  48 (mantel haenszel or peto or der simonian or dersimonian or fixed effect* or latin square*).ti,ab,kf. 39105  49 (met analy* or metanaly* or technology assessment* or HTA or HTAs or technology overview* or technology appraisal*).ti,ab,kf. 13286  50 (meta regression* or metaregression*).ti,ab,kf. 16862  51 (meta-analy* or metaanaly* or systematic review* or biomedical technology assessment* or bio-medical technology assessment*).mp,hw. 532240  52 (medline or cochrane or pubmed or medlars or embase or cinahl).ti,ab,hw. 391722  53 (cochrane or (health adj2 technology assessment) or evidence report).jw. 22002  54 (comparative adj3 (efficacy or effectiveness)).ti,ab,kf. 19430  55 (outcomes research or relative effectiveness).ti,ab,kf. 11863  56 ((indirect or indirect treatment or mixed-treatment or bayesian) adj3 comparison*).ti,ab,kf. 4709  57 (multi* adj3 treatment adj3 comparison*).ti,ab,kf. 313  58 (mixed adj3 treatment adj3 (meta-analy* or metaanaly*)).ti,ab,kf. 181  59 umbrella review*.ti,ab,kf. 2157  60 (multi* adj2 paramet* adj2 evidence adj2 synthesis).ti,ab,kf. 14  61 (multiparamet* adj2 evidence adj2 synthesis).ti,ab,kf. 19  62 (multi-paramet* adj2 evidence adj2 synthesis).ti,ab,kf. 12  63^[[4]](#footnote-4)^ or/41-62 780944  64 40 and 63 325 |
| --- |
| <https://proxy.queensu.ca/login?url=http://ovidsp.ovid.com?T=JS&NEWS=N&PAGE=main&SHAREDSEARCHID=2tnTMnf3rS1cVEW0h6Ks3ooRyMTmgPsbzNUproUeglaMExpZ5jLbbfnctUAFpQ0Gh>  **Embase Classic+Embase <1947 to 2024 August 08>**  1 ((disabled adj1 pe*) or disab*).mp. or exp disabled person/ or exp physically disabled person/ 565718  2 (Amputee* or (hearing adj1 impaired pe*) or paraplegi* or quadriplegi* or "hearing loss" or blindness or (vision adj1 disorder$)).mp. 252929  3 ((developmental adj1 (disabilit* or disorder*)) or ((mental or mentally) adj1 retard*)).mp. or exp developmental disorder/ or exp mental deficiency/ 259994  4 (dependent adj1 ambulation).mp. or walking difficulty/ 17571  5 (mobility adj1 limitation$).mp. or Mobility limitation/ 16693  6 (((self-help adj1 devices) or assistive) adj1 technology).mp. or Self-Help Devices/ 8628  7 (((functional or activity or participation) adj1 limitation*) or ((mobility or vision or hearing or cognitiv*) adj1 impair*) or (intellectual adj1 disabilit*)).mp. [mp=title, abstract, heading word, drug trade name, original title, device manufacturer, drug manufacturer, device trade name, keyword heading word, floating subheading word, candidate term word] 335697  8 exp spinal cord injury/ or ("spinal cord injur*" or SCI).ti,ab,kw. 142976  9 "cerebral palsy".mp. or cerebral palsy/ 56130  10 "spina bifida".mp. or exp spinal dysraphism/ 25839  11 "multiple sclerosis".mp. or exp multiple sclerosis/ 189976  12 amyotrophic lateral sclerosis/ or ("amyotrophic lateral sclerosis" or ALS).ti,ab,kw. 78576  13 exp traumatic brain injury/ or "traumatic brain injur*".mp. 93733  14 exp concussion/ or concussion*.mp. 24306  15 exp muscular dystrophy/ or "muscular dystroph*".mp. 66277  16 exp cerebrovascular accident/ or (stroke* or "cerebrovascular accident*" or cva).ti,ab,kw. 654332  17 exp spinal muscular atrophy/ or "spinal muscular atroph*".ab,ti,kw. 77946  18 exp myopathy/ or myopathy.mp. 115866  19 ((sensory adj1 (impairment or limitation)) or "psycho-social disabilit*" or "psychosocial disabilit*").mp. 3766  20 or/1-19 2414248  21^[[5]](#footnote-5)^ juvenile/ or exp adolescent/ or exp child/ or young adult/ or exp postnatal development/ or (pediatric* or paediatric* or (child* not childbearing) or newborn* or congenital* or infan* or baby or babies or neonat* or pre term or preterm* or premature birth or NICU or preschool* or pre school* or kindergarten* or elementary school* or nursery school* or schoolchild* or toddler* or boy or boys or girl* or middle school* or pubescen* or juvenile* or teen* or youth* or high school* or adolesc* or prepubesc* or pre pubesc*).mp. or (child* or adolesc* or pediat* or paediat*).jn. or (college or universit* or post-secondary).ab,ti. 7571118  22^[[6]](#footnote-6)^ (recreational park/ or (outside or outdoor* or play ground* or playground* or yard* or (school adj ground*) or green space* or greenspace* or blue space* or bluespace* or wilderness or natur* or forest* or park or parks or back yard* or backyard* or environment* or land-based or water-based or beach or bush).ti,ab.) adj1 (Exercise/ or recreation/ or physical activity/ or ((physical adj activity) or active or exercis* or sport* or leisure or recreation* or adventur* or movement or play* or game or games or gym or gyms).ti,ab.) 31176  23 20 and 21 and 22 1035  24 (((systematic* or scoping) adj3 (review* or overview*)) or (methodologic* adj3 (review* or overview*))).ti,ab,kf. 478807  25 ((quantitative adj3 (review* or overview* or synthes*)) or (research adj3 (integrati* or overview*))).ti,ab,kf. 20740  26 ((integrative adj3 (review* or overview*)) or (collaborative adj3 (review* or overview*)) or (pool* adj3 analy*)).ti,ab,kf. 61198  27 (data synthes* or data extraction* or data abstraction*).ti,ab,kf. 56680  28 (handsearch* or hand search*).ti,ab,kf. 14290  29 (mantel haenszel or peto or der simonian or dersimonian or fixed effect* or latin square*).ti,ab,kf. 51935  30 (met analy* or metanaly* or technology assessment* or HTA or HTAs or technology overview* or technology appraisal*).ti,ab,kf. 22620  31 (meta regression* or metaregression*).ti,ab,kf. 20628  32 (meta-analy* or metaanaly* or systematic review* or biomedical technology assessment* or bio-medical technology assessment*).mp,hw. 829249  33 (medline or cochrane or pubmed or medlars or embase or cinahl).ti,ab,hw. 509669  34 (cochrane or (health adj2 technology assessment) or evidence report).jw. 32328  35 (comparative adj3 (efficacy or effectiveness)).ti,ab,kf. 28951  36 (outcomes research or relative effectiveness).ti,ab,kf. 17513  37 ((indirect or indirect treatment or mixed-treatment or bayesian) adj3 comparison*).ti,ab,kf. 8314  38 (multi* adj3 treatment adj3 comparison*).ti,ab,kf. 454  39 umbrella review*.ti,ab,kf. 2285  40 (multi* adj2 paramet* adj2 evidence adj2 synthesis).ti,ab,kf. 35  41 (multiparamet* adj2 evidence adj2 synthesis).ti,ab,kf. 22  42^[[7]](#footnote-7)^ (multi-paramet* adj2 evidence adj2 synthesis).ti,ab,kf. 30  43 meta-analysis/ or systematic review/ or systematic reviews as topic/ or meta-analysis as topic/ or "meta analysis (topic)"/ or "systematic review (topic)"/ or exp technology assessment, biomedical/ or network meta-analysis/ 700702  44 ((systematic* adj3 (review* or overview*)) or (methodologic* adj3 (review* or overview*))).ti,ab,kf. 455350  45 ((quantitative adj3 (review* or overview* or synthes*)) or (research adj3 (integrati* or overview*))).ti,ab,kf. 20740  46 ((integrative adj3 (review* or overview*)) or (collaborative adj3 (review* or overview*)) or (pool* adj3 analy*)).ti,ab,kf. 61198  47 (data synthes* or data extraction* or data abstraction*).ti,ab,kf. 56680  48 (handsearch* or hand search*).ti,ab,kf. 14290  49 (mantel haenszel or peto or der simonian or dersimonian or fixed effect* or latin square*).ti,ab,kf. 51935  50 (met analy* or metanaly* or technology assessment* or HTA or HTAs or technology overview* or technology appraisal*).ti,ab,kf. 22620  51 (meta regression* or metaregression*).ti,ab,kf. 20628  52 (meta-analy* or metaanaly* or systematic review* or biomedical technology assessment* or bio-medical technology assessment*).mp,hw. 829249  53 (medline or cochrane or pubmed or medlars or embase or cinahl).ti,ab,hw. 509669  54 (cochrane or (health adj2 technology assessment) or evidence report).jw. 32328  55 (comparative adj3 (efficacy or effectiveness)).ti,ab,kf. 28951  56 (outcomes research or relative effectiveness).ti,ab,kf. 17513  57 ((indirect or indirect treatment or mixed-treatment or bayesian) adj3 comparison*).ti,ab,kf. 8314  58 (multi* adj3 treatment adj3 comparison*).ti,ab,kf. 454  59 (mixed adj3 treatment adj3 (meta-analy* or metaanaly*)).ti,ab,kf. 263  60 umbrella review*.ti,ab,kf. 2285  61 (multi* adj2 paramet* adj2 evidence adj2 synthesis).ti,ab,kf. 35  62 (multiparamet* adj2 evidence adj2 synthesis).ti,ab,kf. 22  63^[[8]](#footnote-8)^ (multi-paramet* adj2 evidence adj2 synthesis).ti,ab,kf. 30  64 or/24-63 1117139  65 23 and 64 86  66 limit 65 to english language 84 |
| **CINAHL Search**  **Fri, August 9, 2024 10:33:44 AM**   \| **#** \| **Query** \| **Results** \| \| --- \| --- \| --- \| \| S1 \| (MH "Persons with Disabilities+") \| 68,185 \| \| S2 \| (MH "Americans with Disabilities Act") \| 2,899 \| \| S3 \| (MH "Architectural Accessibility+") \| 3,201 \| \| S4 \| (MH "Activities of Daily Living+") \| 81,931 \| \| S5 \| (MH "Self Care+") \| 62,242 \| \| S6 \| (MH "Developmental Disabilities") OR (MH "Child Development Disorders, Pervasive+") OR (MH "Child Development Disorders+") OR (MH "Child Behavior Disorders+") OR (MH "Feeding and Eating Disorders of Childhood+") OR (MH "Intellectual Disability") OR (MH "Learning Disorders+") OR (MH "Motor Skills Disorders") \| 88,460 \| \| S7 \| (MH "Communicative Disorders+") \| 41,157 \| \| S8 \| (MH "Paraplegia+") OR (MH "Quadriplegia+") OR (MH "Hemiplegia") \| 12,498 \| \| S9 \| (MH "Hearing Disorders+") \| 42,741 \| \| S10 \| (MH "Hearing Aids+") \| 20,621 \| \| S11 \| (MH "Rehabilitation of Persons with Hearing Loss+") \| 5,939 \| \| S12 \| (MH "Vision Disorders+") \| 21,566 \| \| S13 \| (MH "Sensory Aids+") \| 20,897 \| \| S14 \| (MH "Assistive Technology") OR (MH "Assistive Technology Devices+") \| 43,136 \| \| S15 \| (MH "Functional Status") \| 32,735 \| \| S16 \| (MH "Mental Disorders+") \| 668,536 \| \| S17 \| TX (disab* OR amputee* OR “functional limitation*” OR "activity limitation*” OR “participation limitation*” OR "activit* of daily living" OR “cognitive* impair*” OR "mental* retard*" OR "mobility limitation*" OR "mobility impairment*" OR "hearing impair*" OR "hearing loss" OR "visual* impair*" OR "vision disorder*" OR "blindness" OR "assistive technolog*" OR "mental disorder*" OR "mental* ill*" person*") \| 463,802 \| \| S18 \| (MH "Spinal Cord Injuries+") \| 23,637 \| \| S19 \| (MH "Amputation+") \| 9,831 \| \| S20 \| (MH "Cerebral Palsy") \| 14,062 \| \| S21 \| (MH "Spina Bifida") \| 2,960 \| \| S22 \| (MH "Multiple Sclerosis+") \| 22,524 \| \| S23 \| (MH "Amyotrophic Lateral Sclerosis") \| 4,715 \| \| S24 \| (MH "Brain Injuries+") \| 34,475 \| \| S25 \| (MH "Brain Concussion+") \| 6,936 \| \| S26 \| (MH "Muscular Dystrophy+") \| 4,371 \| \| S27 \| (MH "Stroke+") \| 81,154 \| \| S28 \| (MH "Attention Deficit Hyperactivity Disorder") \| 19,643 \| \| S29 \| (MH "Muscular Atrophy, Spinal+") \| 940 \| \| S30 \| (MH "Muscular Diseases+") \| 57,350 \| \| S31 \| (MH "Child Development Disorders, Pervasive+") \| 33,109 \| \| S32 \| (MH "Down Syndrome") \| 8,079 \| \| S33 \| TX ("spinal cord injur*" OR Amputation* OR "cerebral palsy" OR "spina bifida" OR "multiple sclerosis" OR "Amyotrophic Lateral Sclerosis" OR "brain injur*" OR "concussion" OR "muscular dystrophy" OR "stroke" OR "cerebrovascular accident*" OR “Attention Deficit Hyperactivity Disorder" OR ADHD OR "spinal muscular atrophy" OR "myopath*" OR "autism" OR autistic OR "down syndrome" OR "peripheral neuropath*") \| 392,502 \| \| S34 \| S1 OR S2 OR S3 OR S4 OR S5 OR S6 OR S7 OR S8 OR S9 OR S10 OR S11 OR S12 OR S13 OR S14 OR S15 OR S16 OR S17 OR S18 OR S19 OR S20 OR S21 OR S22 OR S23 OR S24 OR S25 OR S26 OR S27 OR S28 OR S29 OR S30 OR S31 OR S32 OR S33 \| 1,413,219 \| \| S35 \| ( (pediatric* OR paediatric* OR (child* NOT childbearing) OR newborn* OR congenital* OR infan* OR baby OR babies OR neonat* OR "pre-term" OR preterm OR "premature birth*" OR NICU OR preschool* OR "pre-school*" OR kindergarten* OR kindergarden* OR "elementary school*" OR "nursery school*" OR schoolchild* OR toddler* OR boy OR boys OR girl* OR "middle school*" OR pubescen* OR juvenile* OR teen* OR youth* OR "high school*" OR adolesc* OR prepubesc* OR "pre-pubesc*" OR (MH "Child+") OR (MH "Adolescence+") OR (MH "Minors (Legal)") OR "(MH "Child Abuse, Sexual") OR (MH "Child Behavior Disorders+") OR (MH "Child, Medically Fragile") OR (MH "Child Day Care") OR (MH "Child Behavior+") OR (MH "Child Mortality") OR (MH "Child Passenger Safety") OR (MH "Child Development Disorders, Pervasive+") OR (MH "Child Custody") OR (MH "Child Abuse+") OR (MH "Child Nutritional Physiology+") OR (MH "Child Behavior Checklist") ) OR SO ( child* OR pediatric* OR paediatric* OR adolescent* ) ) \| 1,622,519 \| \| S36 \| (MH "Environment") \| 15,434 \| \| S37 \| TI ( (outside or outdoor* or play ground* or playground* or yard* or (school adj ground*) or green space* or greenspace* or blue space* or bluespace* or wilderness or natur* or forest* or park or parks or back yard* or backyard* or environment* or land-based or water-based or beach or bush) ) OR AB ( (outside or outdoor* or play ground* or playground* or yard* or (school adj ground*) or green space* or greenspace* or blue space* or bluespace* or wilderness or natur* or forest* or park or parks or back yard* or backyard* or environment* or land-based or water-based or beach or bush) ) \| 414,951 \| \| S38 \| (MH "Exercise+") OR (MH "Physical Activity") \| 178,769 \| \| S39 \| (MH "Recreation+") \| 34,511 \| \| S40 \| TI ( ((physical adj activity) or active or exercis* or sport* or leisure or recreation* or adventur* or movement or play* or game or games or gym or gyms) ) OR AB ( ((physical adj activity) or active or exercis* or sport* or leisure or recreation* or adventur* or movement or play* or game or games or gym or gyms) ) \| 587,387 \| \| S41 \| S36 OR S37 \| 420,955 \| \| S42 \| S38 OR S39 OR S40 \| 699,934 \| \| S43 \| S41 N2 S42 \| 69,798 \| \| S44 \| S34 AND S35 AND S43 \| 5,619 \| \| S45 \| (MH "meta analysis" OR MH "systematic review" OR MH "Technology, Medical/EV" OR PT "systematic review" OR PT "meta analysis" OR (((TI systematic* OR AB systematic*) N3 ((TI review* OR AB review*) OR (TI overview* OR AB overview*))) OR ((TI methodologic* OR AB methodologic*) N3 ((TI review* OR AB review*) OR (TI overview* OR AB overview*)))) OR (((TI quantitative OR AB quantitative) N3 ((TI review* OR AB review*) OR (TI overview* OR AB overview*) OR (TI synthes* OR AB synthes*))) OR ((TI research OR AB research) N3 ((TI integrati* OR AB integrati*) OR (TI overview* OR AB overview*)))) OR (((TI integrative OR AB integrative) N3 ((TI review* OR AB review*) OR (TI overview* OR AB overview*))) OR ((TI collaborative OR AB collaborative) N3 ((TI review* OR AB review*) OR (TI overview* OR AB overview*))) OR ((TI pool* OR AB pool*) N3 (TI analy* OR AB analy*))) OR ((TI "data synthes*" OR AB "data synthes*") OR (TI "data extraction*" OR AB "data extraction*") OR (TI "data abstraction*" OR AB "data abstraction*")) OR ((TI handsearch* OR AB handsearch*) OR (TI "hand search*" OR AB "hand search*")) OR ((TI "mantel haenszel" OR AB "mantel haenszel") OR (TI peto OR AB peto) OR (TI "der simonian" OR AB "der simonian") OR (TI dersimonian OR AB dersimonian) OR (TI "fixed effect*" OR AB "fixed effect*") OR (TI "latin square*" OR AB "latin square*")) OR ((TI "met analy*" OR AB "met analy*") OR (TI metanaly* OR AB metanaly*) OR (TI "technology assessment*" OR AB "technology assessment*") OR (TI HTA OR AB HTA) OR (TI HTAs OR AB HTAs) OR (TI "technology overview*" OR AB "technology overview*") OR (TI "technology appraisal*" OR AB "technology appraisal*")) OR ((TI "meta regression*" OR AB "meta regression*") OR (TI metaregression* OR AB metaregression*)) OR (TI meta-analy* OR TI metaanaly* OR TI "systematic review*" OR TI "biomedical technology assessment*" OR TI "bio-medical technology assessment*" OR AB meta-analy* OR AB metaanaly* OR AB "systematic review*" OR AB "biomedical technology assessment*" OR AB "bio-medical technology assessment*" OR MW meta-analy* OR MW metaanaly* OR MW "systematic review*" OR MW "biomedical technology assessment*" OR MW "bio-medical technology assessment*") OR ((TI medline OR AB medline OR MW medline) OR (TI cochrane OR AB cochrane OR MW cochrane) OR (TI pubmed OR AB pubmed OR MW pubmed) OR (TI medlars OR AB medlars OR MW medlars) OR (TI embase OR AB embase OR MW embase) OR (TI cinahl OR AB cinahl OR MW cinahl)) OR (SO Cochrane OR SO health technology assessment OR SO evidence report) OR ((TI comparative OR AB comparative) N3 ((TI efficacy OR AB efficacy) OR (TI effectiveness OR AB effectiveness))) OR ((TI "outcomes research" OR AB "outcomes research") OR (TI "relative effectiveness" OR AB "relative effectiveness")) OR (((TI indirect OR AB indirect) OR (TI "indirect treatment" OR AB "indirect treatment") OR (TI mixed-treatment OR AB mixed-treatment) OR (TI bayesian OR AB bayesian)) N3 (TI comparison* OR AB comparison*)) OR ((TI multi* OR AB multi*) N3 (TI treatment OR AB treatment) N3 (TI comparison* OR AB comparison*)) OR ((TI mixed OR AB mixed) N3 (TI treatment OR AB treatment) N3 ((TI meta-analy* OR AB meta-analy*) OR (TI metaanaly* OR AB metaanaly*))) OR (TI "umbrella review*" OR AB "umbrella review*") OR ((TI multi* OR AB multi*) N2 (TI paramet* OR AB paramet*) N2 (TI evidence OR AB evidence) N2 (TI synthesis OR AB synthesis)) OR ((TI multiparamet* OR AB multiparamet*) N2 (TI evidence OR AB evidence) N2 (TI synthesis OR AB synthesis)) OR ((TI multi-paramet* OR AB multi-paramet*) N2 (TI evidence OR AB evidence) N2 (TI synthesis OR AB synthesis)) \| 346,169 \| \| S46 \| TX "scoping review*" \| 16,987 \| \| S47 \| S45 OR S46 \| 350,383 \| \| S48 \| S44 AND S47 \| 439 \| |
| **SPORTDiscus**  **Fri, August 9, 2024 11:24:50 AM**   \| **#** \| **Query** \| **Results** \| \| --- \| --- \| --- \| \| S1 \| DE "DISABILITIES" OR DE "COMMUNICATIVE disorders" OR DE "DEVELOPMENTAL disabilities" OR DE "HEARING disorders" OR DE "MENTAL illness" OR DE "MOVEMENT disorders" OR DE "PERCEPTUAL disorders" OR DE "VISION disorders" OR DE "PEOPLE with disabilities" \| 26,644 \| \| S2 \| DE "PEOPLE with disabilities" OR DE "PEOPLE with intellectual disabilities" OR DE "WOMEN with disabilities" OR DE "PEOPLE with visual disabilities" OR DE "CHILDREN with disabilities" OR DE "ATHLETES with disabilities" OR DE "STUDENTS with disabilities" \| 23,241 \| \| S3 \| DE "PARAPLEGIA" OR DE "PEOPLE with paraplegia" OR DE "QUADRIPLEGIA" OR DE "PEOPLE with quadriplegia" \| 2,569 \| \| S4 \| TX (disab* OR amputee* OR “functional limitation*” OR "activity limitation*” OR “participation limitation*” OR "activit* of daily living" OR “cognitive* impair*” OR "mental* retard*" OR "mobility limitation*" OR "mobility impairment*" OR "hearing impair*" OR "hearing loss" OR "visual* impair*" OR "vision disorder*" OR "blindness" OR "assistive technolog*" OR "mental disorder*" OR "mental* ill*" person*") \| 85,343 \| \| S5 \| DE "SPINAL cord injuries" \| 5,952 \| \| S6 \| (DE "AMPUTEES") OR (DE "AMPUTEES" OR DE "WOMEN amputees") \| 2,216 \| \| S7 \| DE "CEREBRAL palsy" OR DE "PEOPLE with cerebral palsy" \| 3,609 \| \| S8 \| DE "SPINA bifida" \| 294 \| \| S9 \| DE "MULTIPLE sclerosis" \| 2,518 \| \| S10 \| DE "AMYOTROPHIC lateral sclerosis" \| 442 \| \| S11 \| TX ("spinal cord injur*" OR Amputation* OR "cerebral palsy" OR "spina bifida" OR "multiple sclerosis" OR "Amyotrophic Lateral Sclerosis" OR "brain injur*" OR "concussion" OR "muscular dystrophy" OR "stroke" OR "cerebrovascular accident*" OR “Attention Deficit Hyperactivity Disorder" OR ADHD OR "spinal muscular atrophy" OR "myopath*" OR "autism" OR autistic OR "down syndrome" OR "peripheral neuropath*") \| 67,299 \| \| S12 \| DE "BRAIN injuries" OR DE "BRAIN concussion" OR DE "BRAIN damage" OR DE "CHRONIC traumatic encephalopathy" \| 7,917 \| \| S13 \| DE "MUSCULAR dystrophy" \| 278 \| \| S14 \| DE "STROKE" \| 5,222 \| \| S15 \| DE "ATTENTION-deficit hyperactivity disorder" OR DE "CHILDREN with attention-deficit hyperactivity disorder" \| 1,189 \| \| S16 \| DE "MUSCULAR atrophy" OR DE "POSTPOLIOMYELITIS syndrome" \| 1,082 \| \| S17 \| DE "DOWN syndrome" \| 717 \| \| S18 \| DE "MENTAL illness" OR DE "SCHIZOPHRENIA" OR DE "PEOPLE with mental illness" \| 4,610 \| \| S19 \| DE "DEAF people" OR DE "DEAFBLIND people" OR DE "DEAFBLIND children" OR DE "DEAF athletes" OR DE "DEAFNESS" OR DE "HEARING impaired" OR DE "DEAF people" \| 855 \| \| S20 \| DE "BLIND people" OR DE "DEAFBLIND people" OR DE "DEAFBLIND children" OR DE "BLIND athletes" OR DE "PEOPLE with visual disabilities" OR DE "BLINDNESS" \| 1,389 \| \| S21 \| S1 OR S2 OR S3 OR S4 OR S5 OR S6 OR S7 OR S8 OR S9 OR S10 OR S11 OR S12 OR S13 OR S14 OR S15 OR S16 OR S17 OR S18 OR S19 OR S20 \| 142,916 \| \| S22 \| (MH "Environment") \| 11,415 \| \| S23 \| TI ( (outside or outdoor* or play ground* or playground* or yard* or (school adj ground*) or green space* or greenspace* or blue space* or bluespace* or wilderness or natur* or forest* or park or parks or back yard* or backyard* or environment* or land-based or water-based or beach or bush) ) OR AB ( (outside or outdoor* or play ground* or playground* or yard* or (school adj ground*) or green space* or greenspace* or blue space* or bluespace* or wilderness or natur* or forest* or park or parks or back yard* or backyard* or environment* or land-based or water-based or beach or bush) ) \| 172,443 \| \| S24 \| (MH "Exercise+") OR (MH "Physical Activity") \| 34,175 \| \| S25 \| (MH "Recreation+") \| 13 \| \| S26 \| TI ( ((physical adj activity) or active or exercis* or sport* or leisure or recreation* or adventur* or movement or play* or game or games or gym or gyms) ) OR AB ( ((physical adj activity) or active or exercis* or sport* or leisure or recreation* or adventur* or movement or play* or game or games or gym or gyms) ) \| 895,756 \| \| S27 \| S22 OR S23 \| 178,317 \| \| S28 \| S24 OR S25 OR S26 \| 909,778 \| \| S29 \| S27 AND S28 \| 75,601 \| \| S30 \| S21 AND S29 \| 4,827 \| \| S31 \| DE "SPORTS for people with disabilities" OR DE "ARCHERY for people with disabilities" OR DE "BASKETBALL for people with disabilities" OR DE "BOATING for people with disabilities" OR DE "CRICKET for people with disabilities" OR DE "CYCLING for people with disabilities" OR DE "DIVING for people with disabilities" OR DE "DOGSLEDDING for people with disabilities" OR DE "FISHING for people with disabilities" OR DE "FOOTBALL for people with disabilities" OR DE "GOLF for people with disabilities" OR DE "GYMNASTICS for people with disabilities" OR DE "HANDBALL for people with disabilities" OR DE "HANG gliding for people with disabilities" OR DE "HOCKEY for people with disabilities" OR DE "HUNTING for people with disabilities" OR DE "MARTIAL arts for people with disabilities" OR DE "MOTORCYCLING for people with disabilities" OR DE "MOTORSOCCER" OR DE "PARACHUTING for people with disabilities" OR DE "ROCK climbing for people with disabilities" OR DE "RUGBALL" OR DE "RUNNING for people with disabilities" OR DE "SHOOTING for people with disabilities" OR DE "SKATING for people with disabilities" OR DE "SKIING for people with disabilities" OR DE "SKYDIVING for people with disabilities" OR DE "SNOWSHOEING for people with disabilities" OR DE "SOCCER for people with disabilities" OR DE "SOFTBALL for people with disabilities" OR DE "SPORTS for children with disabilities" OR DE "SPORTS for people with intellectual disabilities" OR DE "SPORTS for people with visual disabilities" OR DE "SURFING for people with disabilities" OR DE "SWIMMING for people with disabilities" OR DE "TENNIS for people with disabilities" OR DE "VOLLEYBALL for people with disabilities" OR DE "WEIGHT training for people with disabilities" OR DE "WHEELCHAIR sports" OR DE "WINDSURFING for people with disabilities" OR DE "WORLD Games for the Deaf" \| 6,840 \| \| S32 \| S27 AND S31 \| 364 \| \| S33 \| S30 OR S32 \| 4,940 \| \| S34 \| ( (pediatric* OR paediatric* OR (child* NOT childbearing) OR newborn* OR congenital* OR infan* OR baby OR babies OR neonat* OR "pre-term" OR preterm OR "premature birth*" OR NICU OR preschool* OR "pre-school*" OR kindergarten* OR kindergarden* OR "elementary school*" OR "nursery school*" OR schoolchild* OR toddler* OR boy OR boys OR girl* OR "middle school*" OR pubescen* OR juvenile* OR teen* OR youth* OR "high school*" OR adolesc* OR prepubesc* OR "pre-pubesc*" OR SO ( child* OR pediatric* OR paediatric* OR adolescent* ) ) \| 270,239 \| \| S35 \| TX ("young adult*" OR college* OR universit* OR post-sceondary OR postsecondary) \| 756,630 \| \| S36 \| DE "CHILDREN" OR DE "AIDS & children" OR DE "BOYS" OR DE "CHILD acrobats" OR DE "CHILD circus performers" OR DE "CHILD dancers" OR DE "CHILD development" OR DE "DANCE for children" OR DE "DEAFBLIND children" OR DE "GIRLS" OR DE "OUTDOOR recreation for children" OR DE "OVERWEIGHT children" OR DE "SCHOOL children" OR DE "SELF-defense for children" OR DE "VIDEO games & children" \| 57,974 \| \| S37 \| S34 OR S35 OR S36 \| 906,871 \| \| S38 \| S33 AND S37 \| 3,354 \| \| S39 \| ((TI systematic* OR AB systematic*) N3 ((TI review* OR AB review*) OR (TI overview* OR AB overview*))) OR ((TI methodologic* OR AB methodologic*) N3 ((TI review* OR AB review*) OR (TI overview* OR AB overview*)))) OR (((TI quantitative OR AB quantitative) N3 ((TI review* OR AB review*) OR (TI overview* OR AB overview*) OR (TI synthes* OR AB synthes*))) OR ((TI research OR AB research) N3 ((TI integrati* OR AB integrati*) OR (TI overview* OR AB overview*)))) OR (((TI integrative OR AB integrative) N3 ((TI review* OR AB review*) OR (TI overview* OR AB overview*))) OR ((TI collaborative OR AB collaborative) N3 ((TI review* OR AB review*) OR (TI overview* OR AB overview*))) OR ((TI pool* OR AB pool*) N3 (TI analy* OR AB analy*))) OR ((TI "data synthes*" OR AB "data synthes*") OR (TI "data extraction*" OR AB "data extraction*") OR (TI "data abstraction*" OR AB "data abstraction*")) OR ((TI handsearch* OR AB handsearch*) OR (TI "hand search*" OR AB "hand search*")) OR ((TI "mantel haenszel" OR AB "mantel haenszel") OR (TI peto OR AB peto) OR (TI "der simonian" OR AB "der simonian") OR (TI dersimonian OR AB dersimonian) OR (TI "fixed effect*" OR AB "fixed effect*") OR (TI "latin square*" OR AB "latin square*")) OR ((TI "met analy*" OR AB "met analy*") OR (TI metanaly* OR AB metanaly*) OR (TI "technology assessment*" OR AB "technology assessment*") OR (TI HTA OR AB HTA) OR (TI HTAs OR AB HTAs) OR (TI "technology overview*" OR AB "technology overview*") OR (TI "technology appraisal*" OR AB "technology appraisal*")) OR ((TI "meta regression*" OR AB "meta regression*") OR (TI metaregression* OR AB metaregression*)) OR (TI meta-analy* OR TI metaanaly* OR TI "systematic review*" OR TI "biomedical technology assessment*" OR TI "bio-medical technology assessment*" OR AB meta-analy* OR AB metaanaly* OR AB "systematic review*" OR AB "biomedical technology assessment*" OR AB "bio-medical technology assessment*" OR MW meta-analy* OR MW metaanaly* OR MW "systematic review*" OR MW "biomedical technology assessment*" OR MW "bio-medical technology assessment*") OR ((TI medline OR AB medline OR MW medline) OR (TI cochrane OR AB cochrane OR MW cochrane) OR (TI pubmed OR AB pubmed OR MW pubmed) OR (TI medlars OR AB medlars OR MW medlars) OR (TI embase OR AB embase OR MW embase) OR (TI cinahl OR AB cinahl OR MW cinahl)) OR (SO Cochrane OR SO health technology assessment OR SO evidence report) OR ((TI comparative OR AB comparative) N3 ((TI efficacy OR AB efficacy) OR (TI effectiveness OR AB effectiveness))) OR ((TI "outcomes research" OR AB "outcomes research") OR (TI "relative effectiveness" OR AB "relative effectiveness")) OR (((TI indirect OR AB indirect) OR (TI "indirect treatment" OR AB "indirect treatment") OR (TI mixed-treatment OR AB mixed-treatment) OR (TI bayesian OR AB bayesian)) N3 (TI comparison* OR AB comparison*)) OR ((TI multi* OR AB multi*) N3 (TI treatment OR AB treatment) N3 (TI comparison* OR AB comparison*)) OR ((TI mixed OR AB mixed) N3 (TI treatment OR AB treatment) N3 ((TI meta-analy* OR AB meta-analy*) OR (TI metaanaly* OR AB metaanaly*))) OR (TI "umbrella review*" OR AB "umbrella review*") OR ((TI multi* OR AB multi*) N2 (TI paramet* OR AB paramet*) N2 (TI evidence OR AB evidence) N2 (TI synthesis OR AB synthesis)) OR ((TI multiparamet* OR AB multiparamet*) N2 (TI evidence OR AB evidence) N2 (TI synthesis OR AB synthesis)) OR ((TI multi-paramet* OR AB multi-paramet*) N2 (TI evidence OR AB evidence) N2 (TI synthesis OR AB synthesis)) \| 31,332 \| \| S40 \| TX (scoping N3 review*) \| 1,482 \| \| S41 \| S39 OR S40 \| 31,959 \| \| S42 \| (S39 OR S40) AND (S38 AND S41) \| 231 \| |
| **Web of Science**  Database: Web of Science Core Collection  Date Run: Fri Aug 09 2024 11:58:46 GMT-0400 (Eastern Daylight Time)  # Entitlements:  - WOS.SCI: 1900 to 2024  - WOS.AHCI: 1975 to 2024  - WOS.ESCI: 2019 to 2024  - WOS.ISTP: 1990 to 2024  - WOS.SSCI: 1900 to 2024  - WOS.ISSHP: 1990 to 2024  # Searches:   \| 1 \| ALL=((disab* OR amputee* OR "activit* of daily living" OR "mental* retard*" OR "hearing loss" OR "developmental disabilit*" OR "dependent ambulation" OR parapleg* OR quadripleg* OR "vision disorder*" OR "blindness" OR "assistive technolog*" OR "mental disorder*" OR "mental* ill*" OR "spinal cord injur*" OR amputation* OR "cerebral palsy" OR "spina bifida" OR "multiple sclerosis" OR "Amyotrophic Lateral Sclerosis" OR "brain injur*" OR "concussion" OR "muscular dystrophy" OR "cerebrovascular accident*" OR "Attention Deficit Hyperactivity Disorder" OR ADHD OR "spinal muscular atrophy" OR "myopath*" OR "autism" OR autistic OR "down syndrome" OR "peripheral neuropath*" OR "intellectual disabilit*" )) \| 1606655 \| \| --- \| --- \| --- \| \| 2 \| ALL=((pediatric* or paediatric* or child* or newborn* or congenital* or infan* or preschool* or pre-school* or kindergarten* or "elementary school*" or "nursery school*" or "day care*" or schoolchild* or toddler* or boy or boys or girl* or "middle school*" or pubescen* or juvenile* or teen* or youth* or "high school*" or adolesc* or pre-pubesc* or prepubesc* or "young adult*")) \| 5637959 \| \| 3 \| ALL=(( outside OR outdoor* OR playground* OR yard* OR greenspace* OR bluespace* OR wilderness OR natur* OR forest* OR park OR parks OR backyard* OR environment* OR land-based OR water-based OR beach OR bush ) ) \| 16132693 \| \| 4 \| ALL=(active OR exercis* OR sport* OR leisure OR recreation* OR adventur* OR movement OR game OR games OR gym OR gyms OR (physical AND ( activit* OR inactivit* )) OR fitness OR accelerometr* OR actigraphy OR "step count*" OR pedomet*) \| 6079006 \| \| 5 \| 4 AND #3 AND #2 AND #1 \| 12726 \| \| 6 \| 4 AND #3 AND #2 AND #1 and Review Article (Document Types \| 1570 \| \| 7 \| 4 AND #3 AND #2 AND #1 and Review Article (Document Types) and English (Languages) \| 1541 \| |
| **Scopus**  ((KEY(( "systematic review*" OR "meta-analysis" OR "technology assessment*" OR "scoping review*" OR "systematic overview*" OR "scoping overview*" OR "methodologic* review*" OR "methodologic* overview*" OR "quantitative review*" OR "quantitative synthes*" OR "integrative review*" OR "collaborative review*" OR "data synthes*" OR "data extraction*" OR "data abstraction*" OR handsearch* OR "hand search*" OR metanaly* OR hta OR htas OR "technology overview*" OR "technology appraisal*" OR "meta regression*" OR metaregression* OR "umbrella review*" OR " meta aggregation" OR "evidence synthesis" OR "knowledge synthesis" ))) OR (ABS(( "systematic review*" OR "meta-analysis" OR "technology assessment*" OR "scoping review*" OR "systematic overview*" OR "scoping overview*" OR "methodologic* review*" OR "methodologic* overview*" OR "quantitative review*" OR "quantitative synthes*" OR "integrative review*" OR "collaborative review*" OR "data synthes*" OR "data extraction*" OR "data abstraction*" OR handsearch* OR "hand search*" OR metanaly* OR hta OR htas OR "technology overview*" OR "technology appraisal*" OR "meta regression*" OR metaregression* OR "umbrella review*" OR " meta aggregation" OR "evidence synthesis" OR "knowledge synthesis" ))) OR (TITLE(("systematic review*" OR "meta-analysis" OR "technology assessment*" OR "scoping review*" OR "systematic overview*" OR "scoping overview*" OR "methodologic* review*" OR "methodologic* overview*" OR "quantitative review*" OR "quantitative synthes*" OR "integrative review*" OR "collaborative review*" OR "data synthes*" OR "data extraction*" OR "data abstraction*" OR handsearch* OR "hand search*" OR metanaly* OR hta OR htas OR "technology overview*" OR "technology appraisal*" OR "meta regression*" OR metaregression* OR "umbrella review*" OR " meta aggregation" OR "evidence synthesis" OR "knowledge synthesis")))) AND ((TITLE-ABS-KEY(( pediatric* OR paediatric* OR ( child* AND NOT childbearing ) OR newborn* OR congenital* OR infan* OR baby OR babies OR neonat* OR "pre-term" OR preterm OR "premature birth*" OR nicu OR preschool* OR "pre-school*" OR kindergarten* OR kindergarden* OR "elementary school*" OR "nursery school*" OR schoolchild* OR toddler* OR boy OR boys OR girl* OR "middle school*" OR pubescen* OR juvenile* OR teen* OR youth* OR "high school*" OR adolesc* OR prepubesc* OR "pre-pubesc*" or "young adult" OR college* OR universit* OR post-secondary OR postsecondary)) OR SRCTITLE(( child* OR pediatric* OR paediatric* OR adolescent )))) AND ((TITLE-ABS-KEY(( physical AND activit* ) OR exercis* OR (physical AND inactivit*) OR fitness OR accelerometr* OR actigraphy OR "step count*" OR pedomet*) AND TITLE-ABS-KEY((outside OR outdoor* OR playground* OR yard* OR greenspace* OR bluespace* OR wilderness OR natur* OR forest* OR park OR parks OR backyard* OR environment* OR land-based OR water-based OR beach OR bush) w/2 ( active OR exercis* OR sport* OR leisure OR recreation* OR adventur* OR movement OR play* OR game OR games OR gym OR gyms )))) AND (TITLE-ABS-KEY((disab* OR amputee* OR "functional limitation*" OR "activity limitation*" OR "participation limitation*" OR "activit* of daily living" OR "cognitive* impair*" OR "mental* retard*" OR "mobility limitation*" OR "mobility impairment*" OR "hearing impair*" OR "hearing loss" OR "visual* impair*" OR "developmental Disabilit*" OR "dependent ambulation" OR parapleg* OR quadripleg* OR "mobility limitation*" OR "vision disorder*" OR "blindness" OR "assistive technolog*" OR "mental disorder*" OR "mental* ill*" OR "spinal cord injur*" OR amputation* OR "cerebral palsy" OR "spina bifida" OR "multiple sclerosis" OR "Amyotrophic Lateral Sclerosis" OR "brain injur*" OR "concussion" OR "muscular dystrophy" OR "stroke" OR "cerebrovascular accident*" OR "Attention Deficit Hyperactivity Disorder" OR ADHD OR "spinal muscular atrophy" OR "myopath*" OR "autism" OR autistic OR "down syndrome" OR "peripheral neuropath*" OR "intellectual disabilit*" ))) |

# **Supplementary Table 3. Database search records.**

| **Scholarly articles** | |
| --- | --- |
| **Search date** | July 2024 |
| **Search database** |  |
| MEDLINE | 325 |
| Web of Science | 1,541 |
| EMBASE | 84 |
| CINAHL | 439 |
| SPORTDiscus | 231 |
| Scopus | 89 |
| Total number of records | 2,709 |
| Total number of records after removing duplicates in Covidence | 2,242 |

# **Supplementary Table 4a. Critical appraisal for systematic reviews (n = 10).**

| **Systematic reviews (n = 10)** | |
| --- | --- |
| **Author/year** | **Item** |

|  | 1 | 2 | 3 | 4 | 5 | 6 | 7 | 8 | 9 | 10 | 11 |
| --- | --- | --- | --- | --- | --- | --- | --- | --- | --- | --- | --- |
| Coussens 2020 | yes | yes | yes | yes | yes | yes | yes | yes | yes | yes | yes |
| Crawford 2014 | yes | yes | yes | yes | yes | yes | yes | yes | yes | yes | yes |
| Gately 2023 | yes | yes | yes | yes | yes | yes | yes | no | yes | yes | yes |
| Graham 2018 | yes | yes | yes | yes | yes | yes | yes | yes | yes | yes | yes |
| Hospodar 2023 | yes | yes | yes | yes | yes | U | no | U | no | yes | yes |
| Kuhaneck 2020 | yes | yes | yes | yes | yes | U | yes | yes | yes | yes | yes |
| Laverdure 2021 | yes | yes | yes | yes | yes | U | U | yes | yes | yes | yes |
| Luckett 2007 | yes | yes | yes | no | no | N/A | U | yes | no | yes | yes |
| Pashmdarfard 2021 | yes | yes | yes | yes | yes | yes | no | yes | yes | yes | yes |
| Sterman 2016 | yes | yes | yes | yes | yes | U | yes | yes | yes | yes | yes |

| Joanna Briggs Institute (JBI) appraisal checklist for systematic reviews and research syntheses was used.  Item 1. Is the review question clearly and explicitly stated?  Item 2. Were the inclusion criteria appropriate for the review question?  Item 3. Was the search strategy appropriate?  Item 4. Were the sources and resources used to search for studies adequate?  Item 5. Were the criteria for appraising studies appropriate?  Item 6. Was critical appraisal conducted by two or more reviewers independently?  Item 7. Were there methods to minimize errors in data extraction?  Item 8. Were the methods used to combine studies appropriate?  Item 9. Was the likelihood of publication bias assessed?  Item 10. Were recommendations for policy and/or practice supported by the reported data?  Item 11. Were the specific directives for new research appropriate?  N/A = Not applicable; U = unclear. |
| --- |

# **Supplementary Table 4b. Critical appraisal for scoping reviews (n = 8).**

| **Scoping reviews (n = 8)** | |
| --- | --- |
| **Author/year** | **Item** |

|  | 1 | 2 | 3 | 4 | 5 | 6 | 7 | 8 | 9 | 10 | 11 |
| --- | --- | --- | --- | --- | --- | --- | --- | --- | --- | --- | --- |
| Anaby 2013 | yes | yes | yes | yes | N/A | N/A | yes | yes | yes | yes | yes |
| Askari 2015 | yes | yes | yes | yes | N/A | N/A | yes | yes | yes | yes | yes |
| Brown 2021 | yes | yes | yes | yes | NA | N/A | yes | yes | yes | yes | yes |
| Dabiri Golchin 2024 | yes | yes | yes | yes | N/A | N/A | yes | yes | yes | yes | yes |
| Huus 2021 | yes | yes | yes | yes | N/A | N/A | yes | yes | yes | yes | yes |
| Jung 2013 | yes | yes | yes | yes | N/A | N/A | no | yes | yes | yes | yes |
| Morgenthaler 2023 | yes | yes | yes | yes | N/A | N/A | yes | yes | yes | yes | yes |
| Therrien 2022 | yes | yes | yes | yes | N/A | N/A | yes | yes | yes | yes | yes |

| Joanna Briggs Institute (JBI) appraisal checklist for systematic reviews and research syntheses was used.  Item 1. Is the review question clearly and explicitly stated?  Item 2. Were the inclusion criteria appropriate for the review question?  Item 3. Was the search strategy appropriate?  Item 4. Were the sources and resources used to search for studies adequate?  Item 5. Were the criteria for appraising studies appropriate?  Item 6. Was critical appraisal conducted by two or more reviewers independently?  Item 7. Were there methods to minimize errors in data extraction?  Item 8. Were the methods used to combine studies appropriate?  Item 9. Was the likelihood of publication bias assessed?  Item 10. Were recommendations for policy and/or practice supported by the reported data?  Item 11. Were the specific directives for new research appropriate?  N/A = Not applicable; U = unclear. |
| --- |

# **Supplementary Table 5. Definition, perception, and/or experiences of active play among children and youth with disabilities and their adult facilitators (n = 10).**

| Author (year) | Perspective | Age in years (mean/median) | Play Type | Conceptualization of play | Evidence related to definition, perception and/or experience(s) of play |
| --- | --- | --- | --- | --- | --- |
| Askari et al. (2015) | Children and youth with ASD | 5–17 (11) years | Play, recreation, leisure, participation, hobbies | Perception | - Parents of children with ASD perceived more difficulty and experienced more emotional stress than parents of children without ASD. However, the parents were just as willing to involve their children in community activities (reported in Lam et al., 2010). |
|  |  |  |  | Experience | Children with HFASD engaged more frequently in solitary activities as sensory issues and difficulties with social interaction impacted their ability to successfully participate in peer-based activities (reported in two studies). |
| Brown et al. (2021) | Children with disabilities and their parents/caregivers | Not indicated | Play | Perception | - Parents of children with disabilities reported that their children viewed being on an elevated play structure as more fun than ground-level activities (reported by Stanton-Chapman et al., 2017).  - Children with varied impaired reported a desire for play equipment shaped in recognizable designs (reported in two studies). |
| Coussens et al. (2022) | Parents of children with ADHD, DCD, and/or ASD | 0–6 (3) years | Play, leisure, participation | Perception | - Parents reported that leisure activities of children with ADHD and/or ASD often took place in the garden or bedroom.  - Parents viewed the physical setting at home as less constricting for the participation in leisure and play among children and youth.  - Parents supported the importance of social communication (playing with others and alone) and independence. |
|  |  |  |  | Experience | - Parents often needed to structure the environment to ensure that their child chose activities that were sufficiently challenging. |
| Gately et al. (2023) | Children with disabilities | 3–12 (7.5) years | Play | Experience | - Children with disabilities felt they “belonged in an area” separate from their peers without disabilities.  - Children with mobility impairments frequently faced physical barriers to play on playground that are labeled as “accessible.” |
| Graham et al. (2018) | Children with physical disabilities | 0–18 (9) years | Play, recreation, leisure | Definition | - Play feels positive: Play was described as a positive experience that was fun, happy and good (reported in three studies).  - Children with physical disabilities enjoyed competition within play and the opportunity to compete alongside their peers (reported in three studies). |
|  |  |  |  | Perception | - Children with PD felt “that no one wanted to play with them.” |
|  |  |  |  | Experience | - Children with PD expressed that they must ask permission to play (reported in five studies).  - Play can draw attention towards or away the child’s disability.  - The need for an adult or support staff to help children with PD participate feels normal. |
| Jung et al. (2013) | Children with autism | 0–8 (4) years | Play | Experience | - Children with autism do not spontaneously participate and take a proactive role in play.  - Children with autism face more difficulty interacting with their peers than adults as their peers are less likely to adjust to their play behaviours. |
| Luckett et al. (2007) | Children with autism | Not indicated | Play | Definition | - Play is dependent on the attitude taken by the child themselves.  - Play is intrinsically motivated rather than motivated by an external reward.  - Play is flexible and spontaneous, involving “some freedom from the constraints of reality.” |
| Morgenthaler et al. (2023) | Children with disabilities | 0–12 (6) years | Outdoor play | Perception | - Children with disabilities seek more intense play experiences.  - Children with disabilities wish to make their own choices about what to play (self-directed).  - They value their play time with and without other children and adults.  - They want to feel included when playing on playgrounds.  - Children with disabilities desire play that is fun. |
| Sterman et al. (2016) | Caregivers of children with disabilities | 6–12 (9) years | Outdoor play | Perception | - Caregivers of children with disabilities perceived outdoor play as important for the overall development of children and youth.  - Caregivers perceived all forms of outdoor activity as opportunities for their child to build self-confidence, positive views about outdoor environments, and encourage acceptance by others. |
|  |  |  |  | Experience | - Caregivers’ lack of knowledge or prior experiences of inclusive opportunities for outdoor play had influenced decisions for their children to engage in activities with other children. |
| Therrien et al. (2022) | Children with AAC needs | 0–13 (6.5) years | Play | Experience | - Children with disabilities and limited speech have diverse experiences on the playground.  - Children with AAC often struggled to communicate with peers.  - Many children with AAC observed their peers but did not actively participate in play. |

# **Supplementary Table 6. Facilitators and barriers of active play among children and youth with disabilities (n = 10).**

| Author (year) | Setting | Phenomenon of interest/Problem | Facilitators | Barriers |
| --- | --- | --- | --- | --- |
| Anaby et al. (2013) | Home  Community  School | How does the environment affect the participation of children and youth with disabilities in out-of-school activities? | **Interpersonal**   - Social support of family and friends   - Family support positively influenced participation (reported in 7 studies).  - Parental involvement (e.g., arranging play, vigilance) and parental support promoted social participation and friendships.  - Peer, friend, and classmate support facilitated participation (reported in 5 studies).  **Community**   - Natural and built environment on participation   - Natural environments (plants, animals) positively influenced recreational activities for children with physical disabilities.  - Playground design improvements increased play among children with autism. | **Interpersonal**   - Negative attitudes   - Negative community attitudes were significant barriers to participation for children with disabilities.  - Stigma and bullying, especially for children with cerebral palsy, commonly hindered participation.  **Organization**   - Lack of support from staff and service providers   - Limited participation was linked to a lack of personal assistance, specialists, and staff-provided information (reported in four studies).  **Community**   - Physical environment   - Physical accessibility was a common barrier to participation for children with cerebral palsy.  - Structural issues and lack of equipment in the physical environment hindered leisure participation.  - Physical barriers (natural and built) were frequently associated with reduced after-school activity participation for children with physical disabilities.   - Transportation   - Limited services (e.g., accessible transportation, community programs, and information access) were barriers to participation for children and youth with cerebral palsy.  **Public Policy**   - Policies   - Non-inclusive institutional policies restricted participation for children and youth with disabilities (reported in three studies). |
| Askari et al. (2015) | In a home, community, and out-of-school setting | What current evidence exists relating to leisure participation patterns and determinants of children and youth with ASD? | **Interpersonal**   - Social support   - Support from parents, siblings, peers, and pets facilitated participation in physical activities (reported in 5 papers).  **Organization**  - Schools providing after-school activities helped encourage participation for children with ASD.  **Community**   - Availability of resources and equipment   - The availability and quality of resources, both at home and outside, supported physical activity.  - Direct exercise equipment (e.g., bikes, scooters) and supportive items (e.g., sneakers, fans), as well as features like a playground, swimming pool, or safe walking/running surfaces, facilitated participation for children with ASD (reported in 2 studies).   - Availability of after-school community programs   - Greater participation in activities was linked to the availability of community programs and after-school physical activities, which promoted involvement for youth with ASD.   - Natural environment   - Positive outdoor conditions (e.g., good weather and absence of insects) and the presence of animals/pets facilitated physical activity participation among children with ASD   - Built environment   - Availability of community parks and playgrounds were reported as a facilitator for engaging in physical activities. | **Individual**   - Body functions   - Sensory sensitivity negatively influenced participation, with children with ASD (reported in 2 studies).  - Adolescents with ASD and lower cognitive skills were less likely to engage in social interactions, be invited to activities, or participate in extracurriculars compared to peers with other disabilities.  - Motor skill difficulties limited participation in physical activities, contributing to negative emotions such as frustration, anger, and sadness.  **Interpersonal**   - Parental perception   - Difficulties with motor skills were perceived by the parents as barriers to participation in physical activities after school.  - Parents of children with ASD often viewed leisure and recreation activities as less important than other community activities, such as shopping, dining out, etc.   - Lack of support   - Some parents were unable to supervise their children or let them go outdoors, thus affecting their participation.  - The lack of a peer partner for leisure pursuits was found to limit participation in physical activities.   - Financial constraints   - Financial constraints with the family was found to be a barrier to participation.  **Community**   - Negative attitudes   - Negative community attitudes, including a lack of understanding of autism, were significant barriers to participation.  - Vulnerability and exposure to bullying were barriers for children with ASD to access organized leisure activities   - Lack of equipment   - Lack of equipment or unsafe equipment (e.g., broken rackets, baseball bats, trampolines, or playground items) was identified as a barrier to physical activity participation.  **Public Policy**  - Limited services, such as lack of accessible transportation, community programs, and information, were barriers to participation for children and youth with ASD.  - Parents reported insufficient information from mainstream settings about local leisure facilities.  - A need for improved staff training to better understand the needs of children with ASD and their families was identified. |
| Coussens et al. (2022) | In a home and community setting | According to the perspective of parents, what are the barriers and facilitators of participation of children with ADHD, developmental coordination disorders, and/or ASD? | **Interpersonal**   - Parental perception   - Parents wanted peers to accept their child in free play involving balance and ball skills, despite mobility barriers.  - Parents supported the importance of social communication and functioning (interacting, playing with others, playing alone, understanding, and communication). | **Individual**  - Parents reported that limitations in feed or toileting hindered participation in leisure and play.  **Interpersonal**   - Parental perception   - Parents of children with developmental disabilities identified their restrictive parental habits and educational views as significant barriers of participation.  - Parents were less likely to enrol their child in community or social programmes if their child needed more assistance with activities of daily living than the programme could provide.  - Parents of young children with DD perceived features and resources with daycare or preschool environment to be less supportive of participation for their child. |
| Crawford et al. (2014) | In a childcare center | An examination of strategies that facilitate inclusion in play among children with physical disabilities in childcare centers. | **Interpersonal**  - Adult facilitator (e.g., classroom teacher) played an important role in incorporating children with physical disabilities into play.   - Specific needs and/or preferences of a child   - A customized approach for a child with a disability appears to be an integral part of the adult facilitator’s job.   - Involvement in encouraging play interaction   - It was important for the adult facilitator to encourage and promote play activities for children with disabilities.  - Teachers providing space for children to engage in peer play was identified as an important facilitator.  - In addition, gradually reducing the physical presence of adult facilitators was shown to promote peer interaction effectively.   - Prompting and praise of child   - Prompting and praise are recommended when children participate in play activities in the classroom.  **Organization**   - Physical environment   - The physical environment of childcare centers significantly influenced play activities among children and youth and decisions about peer inclusion.  - Dramatic play areas involving social pretend play were found to enhance meaningful interactions and communication among children, providing opportunities for inclusive play.   - Availability of toys   - Using preferred toys and reinforcing appropriate play behaviors increased toy engagement to levels comparable to typically developing peers for some children.  - Social toys (e.g., dress-up clothes, blocks, dolls) were more likely to promote cooperative play, while isolate toys (e.g., puzzles, books) encouraged solitary or parallel play.  - Social toys naturally created conditions that supported higher levels of play and interaction among children.   - Type of play activity   - Adapting activities to reduce motor demands (e.g., activities suitable for children using wheelchairs) enabled greater participation and fostered inclusive play.  - Reducing physical demands minimized the impact of disabilities, promoting more equitable participation.  - Socio-dramatic play was particularly effective in increasing social interactions and creating inclusive opportunities for all children. |  |
| Gately et al. (2023) | In a playground setting | What literature exists on the relationship between key areas of development and accessible play settings for children with disabilities? |  | **Individual**  - Children with disabilities reported social stigma as a barrier to play and participation (reported in 4 studies).  **Community**  - Children with mobility impairments frequently faced physical barriers to play, despite playgrounds being labeled as accessible (reported in 8 studies).  - Children with mobility impairments encountered physical barriers that prevented access to able-bodied peers, further decreasing opportunities to socialize. |
| Huus et al. (2021) | In all settings | What are the barriers and facilitators to participation in everyday life for children and adolescents with disabilities living in low- and middle-income countries | **Individual**  - **Positive emotions**: A positive perception of their body and its function encouraged participation.  - **Desire for self-regulation**: Motivation to control body shape and maintain a healthy body drove involvement.  - **Mastery of physical activities:** Mastery helped avoid discomfort and supported a healthy lifestyle.  - **Enjoyment and self-confidence:** Fun experiences boosted self-confidence and encouraged continued participation.  - **Skill development:** Learning new skills was a motivating and facilitating factor.  **Interpersonal**  - Family support: A positive family attitude, love, encouragement, and expressions of interest in activities inspired children and adolescents to be active. Building strong alliances with parents was important.  - Friendships: Having supportive friends with positive attitudes who treated them as equals facilitated participation and provided emotional support.  - Professional and societal support: Care from medical professionals, inclusive attitudes from the general population, and supportive healthcare professionals were vital.  **Organization**  - Opportunities and accessibility: Augmentative and alternative communication methods, as well as opportunities to participate in various activities, played a significant role.  - Schools and sports organizations: Building alliances with mainstream schools, ensuring teachers have positive attitudes, and fostering collaboration with sports organizations were crucial.  **Community**  - Careful planning specific to the needs of children and adolescents with disabilities facilitated participation.  - Suitable and inclusive facilities, like access to wheelchairs, promoted independence.  - A supportive environment, including acceptance from family, friends, staff, and the community, played a crucial role, with friends acting as both helpers and guides.  **Public Policy**  - External economic support, such as funding for wheelchairs, facilitated participation. | **Individual**  - **Perceptions of ability**: Children and adolescents often felt limited by their own physical abilities, which hindered their participation in activities.  - **Physical limitations and fear**: They experienced limitations in body function and feared getting injured while participating.  - **Negative emotions**: Feelings of discomfort, vulnerability, embarrassment, and shame were associated with their physical limitations.  - **Fear of being observed**: Some disliked being watched during physical activities.  - **Exclusion**: Being excluded from activities led to disappointment and negative self-perception.  - **Low intrinsic motivation**: A lack of internal drive to participate was also identified as a barrier.  **Interpersonal**  **- Lack of peer connections:** Children and adolescents with disabilities reported lacking friendships in their peer networks.  - **Bullying and harassment:** They were sometimes bullied, verbally insulted, or harassed during physical activities.  - **Perceptions of incompetence**: They felt others viewed them as slow and incapable, with decisions often being made for them.  - **Lack of professional support**: There was insufficient care and support from medical professionals.  **Organization**  - Children and adolescents with disabilities were excluded from school sports and health classes due to a lack of suitable equipment and insufficient peers with similar disabilities to form teams.  **Community**  - Physical environment was not adapted to the children and adolescents’ needs (e.g., uneven playgrounds)  - Physical activities were not adapted to consider persons with disabilities  - Inadequate public transport, poor roads and infrastructure, busy traffic systems, and the lack of wheelchair ramps were barriers in the environment.  - Negative attitudes against children and adolescents with disabilities were reported, where sometimes peers without disabilities did not treat them as equals.  **Public Policy**  - Insufficient policies are in place  - For example, an inclusion policy allowed children with disabilities to attend mainstream schools but lacked requirements for schools to provide necessary adjustments for equal participation.  - Barrier reported on the transportation between school and home. |
| Morgenthaler et al. (2023) | In an outdoor setting | To summarize the perspectives of children with and without disabilities on environmental qualities that enhance their play experiences in community playgrounds | **Individual**  - Children often seek intense and exciting play experiences involving diverse movements and sensory stimulation.  - Children want to make their own choices about what to play by finding suitable challenges, using the environment flexibly, and having moments to unfold their own play.  - Being away from adults and other children revealed a connection with the theme of choices and self-directed play.  - The experience of fun was essential and interwoven in outdoor play among children and youth.  **Community**  - The physical and social environmental characteristics are important in the feelings of connection with the playground among children and youth. Being familiar with the playground, feeling welcomed and safe, and enjoying the playground aesthetics are important to their experience. |  |
| Pashmdarfard et al. (2021) | In a home and community setting | What factors influence the participation of children with cerebral palsy across the eight areas of occupation identified in the Occupational Therapy Practice Framework (OTPF)? | **Individual**   - Motor skills and physical ability   - Motor skills were associated directly with the intensity of participation in play activities; the better the motor skills of the child (physical ability), the greater their participation in sports and non-sports play activities (reported in 6 studies).  - Motor skills (level of physical ability and/or gross motor function) were an important predictor of participation in leisure activities (reported in 11 studies). | **Individual**   - Age   - One study found that participation in leisure activities decreased between primary school age and adolescence in children with cerebral palsy. |
| Sterman et al. (2016) | In an outdoor setting | A qualitative study synthesis on the decision-making processes of caregivers on outdoor play among children with developmental disabilities. | **Individual**  - Children were more motivated to engage in active play when they perceived it as meaningful and had positive inner drive and self-confidence.  - Presenting activities in ways that children found enjoyable or interesting increased their willingness to participate.  **Interpersonal**  - Caregivers must navigate their understanding of how their child can engage in community recreation, including identifying opportunities and fostering motivation for sustained participation in outdoor play.  - Caregivers prioritized decisions they believed were in their child's best interest, often opting out of structured recreation if there was uncertainty about the child's acceptance.  - Caregivers often considered the intrinsic factors of their child when deciding if outdoor play in a particular setting was appropriate. | **Individual**  - Seasonal weather can impact motivation among children and youth to play as well as caregivers’ decisions about supporting their children in outdoor play.  **Interpersonal**  - Caregivers discussed how finances frequently made them more hesitant about allowing their child to participate in outdoor play opportunities (reported in 4 studies).  **Community**  - Accessibility of the built environment was mostly cited as a barrier.  - Playgrounds were reported to be inaccessible to children with disabilities, often requiring adults to physically assist children and restricting the opportunity for unstructured play (reported in 4 studies).  - Negative social attitudes towards the abilities of children with disabilities can affect the decision-making of caregivers on outdoor play (reported in 6 studies). |
| Therrien et al. (2022) | In an outdoor setting | To investigate the playground experiences of children with disabilities, including those with limited speech. | **Interpersonal**  - Support from adults enhanced playground participation among children with AAC (Ripat & Becker, 2012).  **Community**   - Interventions for supporting engagement or communication   - Interventions were effective in increasing peer engagement and communication  - A video self-modeling intervention led to increased initiations, such as holding a peer’s hand (Buggey et al., 2011).  - A social skills program increased verbal interactions for children with limited speech (Hall & Smith, 1996). | **Individual**  - Some children with AAC that had challenging behaviors prevented engagement in peer interactions or play.  **Community**   - Physical accessibility   - Physical inaccessibility of settings like playgrounds and gyms limited the ability of children and youth to leverage their strengths (Egilson & Traustadottir, 2009).   - Usability   - Accessible playgrounds often lacked functional usability. For example, ramps existed, but no play opportunities were available at the top (Ripat & Becker, 2012).   - Safety   - Safety barriers included inadequate shade, which posed challenges for children with difficulty regulating body temperature (Ripat & Becker, 2012).   - Travel to playground   - Sidewalks leading to playgrounds were often poorly maintained, making access difficult for wheelchairs and walkers (Stafford, 2017).  - Public transit was unavailable in some areas, and families often lacked wheelchair-accessible vehicles (Stafford, 2017). |

# **Supplementary Table 7. Effectiveness of interventions targeting active play among children and youth with disabilities (n = 7).**

| Author (year) | Setting | Intervention | Key findings | Quality appraisal tool used and scoring | Quality of studies and effectiveness of interventions |
| --- | --- | --- | --- | --- | --- |
| Brown et al. (2021) | In a playground setting | Inclusive playground design | - Entry points   - Ensure playground entrances are wide and free of obstacles  - Provide wide, flat and firm pathways leading to the playground  - Enclose the playground to prevent children from straying   - Surfacing and paths   - Use a flat uniform surface that consists of material that is moderately firm and stable  - Incorporate ramps that provide access to and between elevated play components   - Features to foster inclusive play   - Implement play equipment that is accessible to all children  - Ensure a variety of play equipment that provides appropriate challenges for children of all ages and abilities  - Provide and spread-out different types of sensory play components across the play space to reduce overstimulation  - Offer solitary play components for escaping overstimulation  - Implement play components shaped in recognizable designs, allowing for creative and imaginative pursuits  - Include features to aid spatial orientation, communication and guidance for using play space  - Provide shaded spaces to aid body temperature regulation   - Staffing and supervision   - The presence of trained staff who can supervise and assist children on playgrounds  - Their presence is important in initiating play   - Design process   - Including playground users in the design process | No quality appraisal conducted | Not applicable |
| Dabiri Golchin et al. (2023) | In a community setting | Active mobility and participation | - Power mobility devices enhance play participation by increasing mobility - Power mobility devices was reported to improve play participation, allowing children to engage in more group play with peers and in less solitary play - Children perceived play using a power mobility device as fun - The use of augmentative and alternative communication (AAC) was reported to improve communication, partnership, and play participation | No quality appraisal conducted due to the design of the study (scoping review); quality appraisal is not a required step of scoping reviews | Not applicable |
| Hospodar et al. (2023) | In a home, hospital, and community setting | Active mobility and participation | - Using MROCs resulted in gains in mobility among children with disabilities.  - Children displayed enjoyment and positive facial expressions and communicative sounds while using MROC.  - MROCs were found to have a positive impact on the participation of children with disabilities, such as social interactions and play behaviors. | Appraised using McMaster critical review form for quantitative studies and the SRQR for qualitative studies  Quality assessed using the Levels of Evidence by the AACPDM; (I-V scale, I = highest evidence, V= lowest evidence) | The study quality and level of evidence varied, with most studies (17 out of 20) being classified as Level IV or V (the lowest levels of evidence) according to AACPDM protocol. Only three studies met Level III criteria and were assigned quality ratings. |
| Jung et al. (2013) | In all settings | Play skills | - Video and live modeling   - Observation and imitation strategies have been effective in teaching various skills to children with autism.  Video modeling (VM) encourages skill acquisition through observational learning by showing videotaped demonstrations of desired behaviors.   - Pivotal response training (PRT)   - PRT has been shown to effectively improve the language, social interactions, and play skills of children with autism.  - PRT is a naturalistic instructional strategy that incorporates the interests of the child into discrete trials in natural environments, following the child’s lead.  - Adults use the favorite toys of the child, model appropriate play, and reinforce the responses of the child while providing exemplars and taking turns.   - Script training   - Script training is used to teach children specific sequences of play behavior.  - One study’s findings reported all children learned the scripts and exhibited more theme-related social interactions. | No quality appraisal conducted  Looked at social validity and procedural integrity, yet did not explicitly state their methods in doing so | Only 8 reported the social validity measures of play skills and 9 studies reported the measurement of procedural integrity. |
| Kuhaneck et al. (2020) | In a home, school, research locations, and summer camp setting | Play | - Interventions targeting play were effective in improving play (reported in 3 RCTs).  - Studies modified the physical play location, changed play partners, adjusted sensory aspects of the environment, used visual aids to enhance performance, or selected highly motivating materials for the child.  - Imitating the child was the most consistently effective strategy in improving play outcomes (reported in 3 RCTs and 4 studies).  - There is moderate to strong evidence that modeling improves functional play skills and the ability to engage in play dialogue (reported in 3 RCTs and 5 studies).  - Moderate evidence suggests that changes to the play environment enhance play performance (reported in 2 RCTs and 5 studies). | Levels of Evidence by the AACPDM; (I-V scale, I = highest evidence, V= lowest evidence) | The quality of evidence varied considerably, with 14 of the studies demonstrating less rigorous designs and a variety of methodological issues. |
| Laverdure et al. (2021) | In a home and community setting | Performance of activities of daily living, play, and leisure | - Supporting engagement in occupations   - Engagement in ADL, play, and leisure occupations and activities was found to improve participation and performance in these areas for children and youth with disabilities.   - Cognitive support for participation   - Occupational participation and performance were found to improve when environments and tasks are adapted to support engagement and when children and youth are provided with explicit occupational skills training (reported in 12 studies).  - Interventions involving collaborative goal setting, occupation-based training and education, and coaching and feedback, can improve ADL, play, and leisure participation and performance in children and youth (reported in 7 studies). | The risk of bias was assessed using the AMSTAR Tool  The strength of evidence was examined using the US Preventative Services Task Force ratings: Strong evidence (Level I) is consistent results are reported across at least two randomized controlled trials (RCTs) and other well-conducted studies; Moderate evidence (Level II) is consistent or slightly inconsistent results are reported in at least one RCT or two or more individual well-conducted studies with lower levels of evidence; Mixed evidence (Level III) is inconsistent results were reported across a number of studies; and insufficient evidence (Level IV) is the number and quality of studies available are insufficient to draw specific conclusions and recommendations. | - Evidence supporting the use of cognitive supports to enhance engagement in play and leisure for children and youth with disabilities is limited and of low strength.  - Six papers were level I (highest quality) including three randomized controlled trials (RCTs), seven papers were level II, and seven papers were level III. |
| Luckett et al. (2007) | In a clinic and school setting | Play skills | - Behavioral approaches can sometimes positively influence disposition toward play among children and youth (reported in 13 articles).  - Successful interventions often included addition features such as developmental approach to build on existing skills and peer support.  - The most effective behavioural interventions to teaching play among children with autism were those that relied on the motivating nature of the activities themselves. Several interventions relied on intrinsic reinforcement from activities or materials rather than extrinsic rewards (reported in 3 articles). | No quality appraisal conducted | Not applicable |
| Abbreviations:  AACPDM: American Academy of Cerebral Palsy and Developmental Medicine; AMSTAR: A Measurement Tool to Assess Systematic Reviews | | | | | |

1. Lines 1-33 based on searches developed by: Ioerger, M., et al. Developing a systematic search strategy related to people with disability: A brief report testing the utility of proposed disability search terms in a search about opioid use. Disabil Health J 2019;12(2): 318-322. and Walsh, ES., Peterson, JJ and Judkins, DZ. Searching for disability in electronic databases of published literature. Disabil Health J 2014;7(1) : 114-118 <https://doi.org/10.1016/j.dhjo.2013.10.005>. [↑](#footnote-ref-1)
2. Line 34 is additional terms suggested by parent partners. [↑](#footnote-ref-2)
3. Line 35 from Joffe A, Anton N, Lequier L, Vandermeer B, Tjosvold L, Larsen B, Hartling L. Nutritional support for critically ill children. Cochrane Database of Systematic Reviews 2009, Issue 2. Art. No.: CD005144. DOI: 10.1002/14651858.CD005144.pub2. and Tjosvold L, Campbell SM, Dorgan M. Filter to Retrieve Pediatric Articles in the OVID Medline Database. Geoffrey & Robyn Sperber Health Sciences Library, University of Alberta. Rev. September 14, 2020. <https://docs.google.com/document/d/1N1-YdflFPmKKy4c3t5LjoYv_90sSzUEkDzPd7_13MDE/edit#heading=h.qi55eeyvgzy9> with additional terms for Young Adults aged 18-21 added. [↑](#footnote-ref-3)
4. Lines 41-62 from SR / MA / HTA / ITC - MEDLINE, Embase, PsycInfo. In: CADTH Search Filters Database. Ottawa: CADTH; 2024: [https://searchfilters.cadth.ca/link/33. Accessed 2024-08-09](https://searchfilters.cadth.ca/link/33.%20Accessed%202024-08-09). With adaptation to include scoping reviews [↑](#footnote-ref-4)
5. Desmeules, R. Filter to Retrieve Pediatric Articles in the OVID EMBASE Database. Geoffrey & Robyn Sperber Health Sciences Library, University of Alberta. Rev. April 20, 2020. <https://docs.google.com/document/d/1N1-YdflFPmKKy4c3t5LjoYv_90sSzUEkDzPd7_13MDE/edit#heading=h.ldbxqb34y1kj> With alterations to include Young Adult [↑](#footnote-ref-5)
6. Line 22 from a search strategy for outdoor play developed by Lindsay Sikora, University of Ottawa [↑](#footnote-ref-6)
7. Lines 24-42 from SIGN Search Filters for Systematic Reviews in Embase: <https://www.sign.ac.uk/using-our-guidelines/methodology/search-filters/> [↑](#footnote-ref-7)
8. Lines 43-63 from adapted from SR / MA / HTA / ITC - MEDLINE, Embase, PsycInfo. In: CADTH Search Filters Database. Ottawa: CADTH; 2024: <https://searchfilters.cadth.ca/link/33>. Accessed 2024-08-09. [↑](#footnote-ref-8)
